# Multiscale heterogeneity of atypical functional connectivity in autism

**DOI:** 10.1038/s44220-026-00656-y

**Published:** 2026-06-01

**Authors:** Iva Ilioska, Marianne Oldehinkel, Alberto Llera, Maroš Rovný, Ting Mei, Seyed Mostafa Kia, Dorothea L. Floris, Julian Tillmann, Rosemary J. Holt, Eva Loth, Tony Charman, Declan G. M. Murphy, Christine Ecker, Tobias Banaschewski, Maarten Mennes, Christian F. Beckmann, Andre Marquand, Jan K. Buitelaar, Alex Fornito

**Affiliations:** 1 Department of Psychiatry, University of Cambridge, Cambridge, UK; 2 Department of Medical Neuroscience, Donders Institute for Brain, Cognition and Behaviour, Radboud University Medical Center, Nijmegen, the Netherlands; 3 MRC CBU, University of Cambridge, Cambridge, UK; 4 Department of Intelligent Systems, Tilburg University, Tilburg, the Netherlands; 5 Methods of Plasticity Research, Department of Psychology, University of Zürich, Zurich, Switzerland; 6 Department of Psychology, Institute of Psychiatry, Psychology and Neuroscience, King’s College London, London, UK; 7 Roche Pharma Research and Early Development, Roche Innovation Center Basel, Basel, Switzerland; 8 Autism Research Centre, Department of Psychiatry, University of Cambridge, Cambridge, UK; 9 Department of Forensic and Neurodevelopmental Sciences, Institute of Psychiatry, Psychology and Neuroscience, King’s College London, London, UK; 10 Department of Child and Adolescent Psychiatry, University Hospital, Goethe University, Frankfurt am Main, Germany; 11 Department of Child and Adolescent Psychiatry, Central Institute of Mental Health, Medical Faculty Mannheim, University of Heidelberg, Mannheim, Germany; 12 Centre for Functional MRI of the Brain, University of Oxford, Oxford, UK; 13 Karakter Child and Adolescent Psychiatry University Center, Nijmegen, the Netherlands; 14 Turner Institute for Brain and Mental Health, School of Psychological Sciences, and and Monash Biomedical Imaging, Monash University, Melbourne, Victoria Australia

**Keywords:** Autism spectrum disorders, Autism spectrum disorders

## Abstract

Group-mean comparisons often identify atypical functional connectivity in autism, but it remains unclear whether these findings consistently manifest at the individual level. Here we use normative modeling to quantify the interindividual heterogeneity of atypical functional connectivity across multiple brain scales using multicenter resting-state functional magnetic resonance imaging data from 1,824 participants (796 autistic individuals and 1,028 neurotypical controls) in a cross-sectional study across 32 sites. We find that no single functional connectivity estimate showed extreme deviation from normative expectations in more than 4% of people in either group. However, these deviations converged on common regions and networks in autistic people, who showed up to double the level of overlap compared with controls. Specifically, autistic participants demonstrated convergent hypoconnectivity in sensorimotor and attention regions and convergent hyperconnectivity between frontoparietal and default mode networks. Functional connectivity deviation patterns significantly predicted social and cognitive abilities. These findings demonstrate that autism exhibits scale-dependent heterogeneity, characterized by normative variability at the connection level but significant convergence at regional and network scales. These convergent regions and networks may be used to identify targets for individualized therapeutic development.

## Abstract (web summary)

Using normative modeling to measure the interindividual variability of atypical brain functional connectivity in autism, the authors identify heterogeneity at the level of specific connections alongside high convergence at regional and network scales. These findings offer a neurobiological framework for understanding both individual differences and shared clinical features in autism.

## Main

Autism affects approximately 1–2% of the global population and is characterized by atypical social interaction and communication, restricted interests, repetitive behaviors and sensory processing differences^1^. These diagnostic criteria point to a common phenotype in autistic people, but neurobiological characteristics and clinical outcomes vary dramatically across individuals^2^, creating substantial challenges for developing personalized treatments and reliable biomarkers.

Autism is widely viewed as a consequence of atypical brain connectivity^3^, but the pervasive heterogeneity of the phenotype has led to a literature replete with inconsistent findings regarding which brain regions are affected and whether connectivity is increased or decreased in autism^3^. Although large-scale studies have identified robust differences at the level of group means^4,5^, the lack of reproducible, person-specific biomarkers means that diagnosis relies entirely on behavioral observation^1^, which is a considerable limitation for early intervention and precision medicine approaches.

This limited progress may result from a reliance on traditional case–control studies, which only compare group means, inadequately capturing individual neurobiological variability^6^. Normative modeling offers a paradigm shift by quantifying how person-specific brain measures deviate from age- and sex-adjusted normative expectations^7,8^, thus allowing for inference at the level of individuals. This approach has revealed highly individualized patterns of deviations in various measures of brain structure and function across psychiatric conditions, suggesting that group averages poorly represent individual patient profiles^8–12^.

The brain is organized across multiple spatial scales, such that specific interregional connections are embedded within regions, which belong to broader functional networks. As such, it is possible that different autistic individuals may display disruptions of distinct connections but that these disrupted connections may nonetheless be concentrated on or within specific regions or networks. A similar principle has been established in autism genetics, where more than 100 identified risk genes linked to distinct molecular mechanisms nonetheless converge on a limited set of biological pathways related, in particular, to synaptic function and transcriptional regulation^13–15^. We propose that this convergence extends to brain organization, such that variable connection-level disruptions may converge on shared regions and networks. Segal et al.^10^ recently demonstrated this phenomenon for person-specific deviations of gray matter volume across psychiatric disorders. Here, we test whether the same scale-dependent convergence characterizes atypical functional connectivity (FC), defined as interregional correlations in resting-state functional magnetic resonance imaging (fMRI) signals, in autism.

**Fig. 1:**
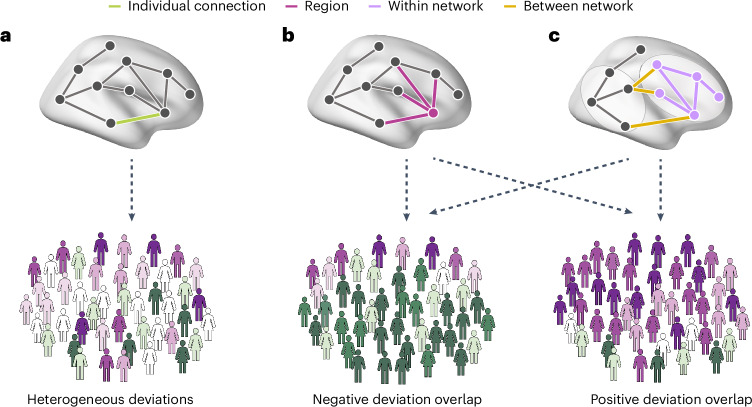
Multiscale heterogeneity of FC deviations in autism. **a**, A connection-level analysis involves quantifying the extent to which each person deviates from model expectations at each interregional FC estimate. We expect there to be little overlap across individuals in terms of the specific connections affected, leading to a heterogeneous profile of connection-level deviations across people. **b**, Despite such heterogeneity, deviations may nonetheless converge on connections linked to specific brain regions. We expect people with autism to show a higher level of interindividual overlap or consistency at this regional level. **c**, FC deviations may also aggregate within specific canonical networks or between specific pairs of networks. Bottom: the greater prevalence of either positive (purple) or negative (green) deviations from model expectations within the autism sample.

We applied cross-sectional normative modeling across three spatial scales (connections, regions and networks) to characterize the interindividual heterogeneity of FC in a large multisite dataset of people with autism and neurotypical controls. We hypothesized that connection-level deviations would be highly heterogeneous, showing minimal overlap among individuals, but that these atypical FC estimates would nonetheless be concentrated within common brain regions and networks. Such scale-dependent heterogeneity represents a viable neurobiological correlate of phenotypic variations (related to high connection-level heterogeneity) and consistencies (related to region- and network-level convergence) in people with autism (Fig. 1).

## Results

### FC deviations are heterogeneous at the connection level

**Fig. 2:**
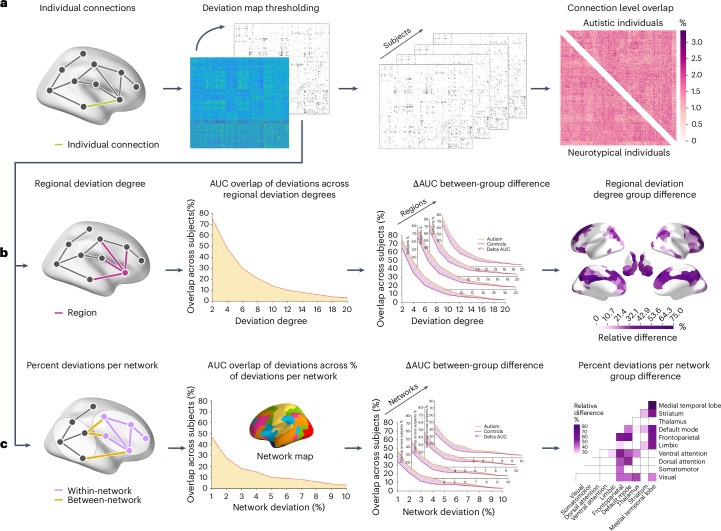
Workflow for quantifying deviation overlap across participants at the levels of connections, regions and networks. **a**, We used normative modeling to generate a deviation matrix for each participant, comprising deviation estimates for 75,855 FC estimates between each pair of 390 regions. A *z*-score threshold of |*z*| > 2.3 (two-sided, *P* = 0.01) was used to identify extreme deviations. The thresholded matrices were subsequently used to calculate the overlap in extreme deviations at each connection across participants. Group differences in connection-level overlap were assessed using 10,000 group-label permutations, with *P* values corrected for multiple comparisons using the FDR_BH_ procedure. **b**, In the region-level analyses, we quantified the regional deviation degree for each participant as the total number of extreme deviant FC estimates attached to each region. We applied thresholds to deviation degree values ranging from 1 to 20 deviant connections and calculated the number of individuals with a deviation degree equal to or exceeding each threshold in each region. We computed the AUC across these thresholds for each region in each group. Group differences in AUC values were tested using two-sided permutation tests (10,000 permutations of diagnostic labels), with *P* values corrected across all regions using FDR_BH_. **c**, A similar approach was used at the network level. We calculated the percentage of extreme FC deviations falling within or between each pair of ten canonical networks. The resulting matrices were thresholded using values between 1% and 10%, and AUC values were compared between groups using two-sided permutation tests (10,000 permutations) with FDR_BH_-corrected *P* values.

**Fig. 3:**
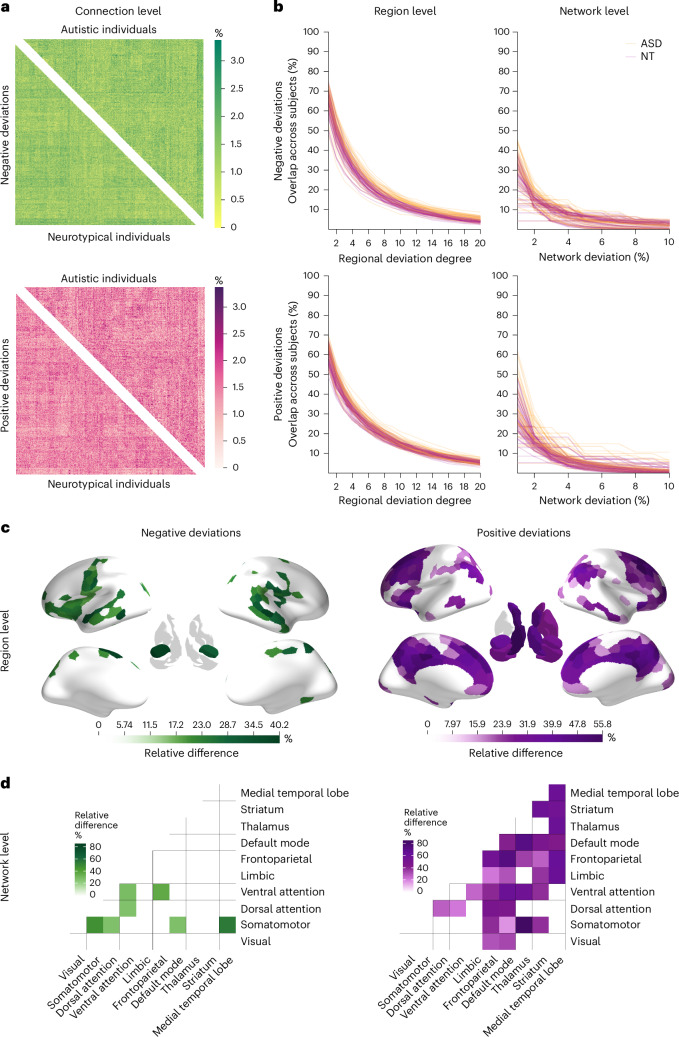
Group differences in deviation overlap at connection, region and network levels. **a**, A connection-level heat map showing the percent overlap of FC deviations across participants (positively deviating—pink; negatively deviating—green). Positive deviations are connections with a larger value than expected on the basis of the normative model, whereas negative deviations are connections that show lower FC than predicted by the normative model. The upper triangle shows the overlap across autistic individuals, whereas the lower triangle shows the overlap across neurotypical controls. **b**, Line plots showing the overlap of participants at the region and network levels. The first column shows the overlap for positive and negative regional deviation degree across 20 thresholds. Each line represents a region in the brain. Yellow color indicates overlap across autistic individuals, whereas red indicates overlap across neurotypical controls. The second column shows the percentage overlap of negative and positive network deviations. Here, each line represents a within or between network overlap across participants, where yellow indicates overlap across the group of autistic individuals, and red indicates the group of controls. **c**,**d**, Regions (**c**) and networks (**d**) with significantly greater overlap in autistic individuals, represented as relative differences. Green color indicates overlap of negative deviations; purple color indicates overlap of positive deviations. Between-group differences are presented as the percentage difference in the overlap between the groups, that is, $\mathrm{relative}\,\mathrm{difference}=\frac{{\mathrm{AUC}}_{\mathrm{autism}}-{\mathrm{AUC}}_{\mathrm{neutotypical}}}{{\mathrm{AUC}}_{\mathrm{neurotypical}}}$. This measure represents the proportional difference in AUC between groups.

To characterize the interindividual heterogeneity of FC in autism, we applied Gaussian process regression normative models to resting-state fMRI data from 1,824 participants (796 autistic and 1,028 neurotypical) across 32 sites. For each participant, 75,855 pairwise FC estimates were modeled as a function of age, sex and mean framewise displacement (FD). FC was mapped using the Schaefer 400 cortical parcellation^16^ and 15 subcortical regions from the Harvard–Oxford atlas^17^ (25 regions excluded for low coverage, leaving 390). Deviations from normative expectations quantified as *z*-scores (Figs. 2a and 3a). Models were trained on neurotypicals and tested on autistic participants, with neurotypical deviations obtained via tenfold cross-validation. Extreme deviations were defined as |*z*| > 2.3 (approximately *P* < 0.01; Methods).

The total number of extreme deviations per participant varied widely in both groups. In autistic individuals, positive deviations ranged from 0 to 5,652 (median of 741.5) and negative deviations from 0 to 5,119 (median of 657); in neurotypical controls, positive deviations ranged from 35 to 3,570 (median of 760) and negative deviations from 105 to 4,745 (median of 673.5). Wilcoxon rank-sum tests revealed no group differences in total deviation burden (*P*_positive_ = 0.086; *P*_negative_ = 0.4), and an independent two-sample *t*-test on positive-to-negative ratios indicated no hyper- or hypoconnectivity bias (*t* = 0.58; *P* = 0.55).

To quantify interindividual overlap, we calculated, for each connection and separately for each group, the percentage of participants showing an extreme deviation (Fig. 2a). Overlap did not exceed 3.4% in either group (Fig. 3a), indicating that no single connection was consistently affected across individuals. Permutation testing (10,000 label permutations with Benjamini–Hochberg false discovery rate (FDR_BH_) correction) revealed no group differences in connection-level overlap (all *P*_FDR_ > 0.05).

### Heterogeneous connection-level deviations converge on common brain regions

Given this connection-level heterogeneity, we next asked whether the affected connections nonetheless converged on common brain regions. For each participant, we computed the deviation degree of each region, which corresponds to the total number of extreme FC deviations (|*z*| > 2.3) attached to that region (Fig. 2b). As deviation degree is continuous, we evaluated overlap across a range of thresholds (1 ≤ *τ* ≤ 20), counting at each threshold the number of participants with a deviation degree of at least *τ*. The resulting area under the curve (AUC) provided a threshold-free summary of regional overlap, which was compared between groups using 10,000 permutations of diagnostic labels with FDR_BH_ correction.

Regional overlap ranged from 41.9% to 77.8% at the lowest threshold (*τ* = 1) and from 1.8% to 12.8% at the highest (*τ* = 20; Fig. 3b). Autistic individuals showed significantly higher overlap than neurotypical controls for negative deviations in sensorimotor, anterior insula, prefrontal, temporal pole, visual and amygdala regions and for positive deviations in medial prefrontal, superior frontal, cingulate, inferior parietal lobule and subcortical areas (Fig. 3c). Neurotypical controls showed no regions with significantly greater overlap than autistic individuals, and results were largely consistent across alternative thresholds (Supplementary Section 9).

### Deviation convergence extends to canonical functional networks

We applied an analogous approach at the level of canonical functional networks. Schaefer regions were assigned to seven cortical networks^18^, with subcortical regions labeled as thalamus, striatum or medial temporal lobe, yielding ten networks. For each participant, we calculated the percentage of extreme deviations falling within and between each network pair (Fig. 2c) and compared AUCs across thresholds (1–10%) between groups using 10,000 permutations with FDR_BH_ correction.

Network-level overlap ranged from 4.4% to 61.9% at the lowest threshold (*τ* = 1) and from 0% to 5.4% at the highest (Fig. 3b). For negative deviations, autistic individuals showed significantly greater overlap than neurotypical controls within the visual and ventral attention networks for connections linking the somatomotor network to other systems (particularly visual, dorsal attention and default mode) and for connections linking medial temporal regions to the somatomotor, ventral attention, limbic and default mode networks (DMN) (Fig. 3d). For positive deviations, autistic individuals showed greater overlap in connections linking the default mode and cognitive control networks with the rest of the brain, as well as in subcortical–subcortical and subcortical–cortical connections. Neurotypical controls did not show significantly greater overlap at any threshold (Supplementary Section 9).

### Sensitivity analyses

To assess robustness, we examined the effects of participant sex, attention-deficit–hyperactivity disorder comorbidity, psychotropic medication and dataset. Results were consistent across all analyses (Supplementary Sections 10 and 12 and Supplementary Tables 4 and 5).

### FC deviations predict clinical and cognitive measures

**Fig. 4:**
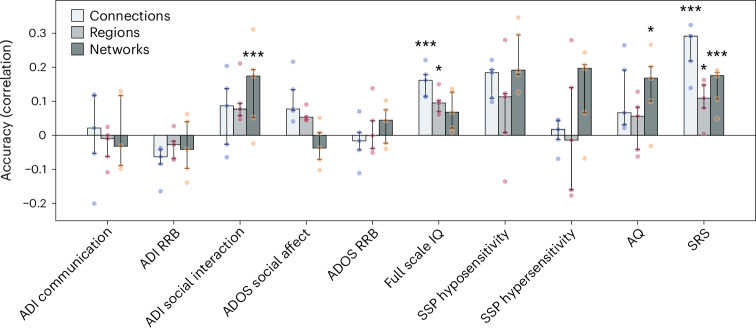
SVR accuracy (Pearson’s *r* between true and predicted values) across levels of FC deviations and behavioral variables. The bars represent median accuracy across five cross-validation folds; error bars represent the interquartile range across folds. The individual data points denote fold-level accuracy. Sample sizes per variable were as follows: ADI-R, *n* = 584 autistic participants; ADOS Social, *n* = 509 autistic participants; ADOS Restrictive and Repetitive Behavior, *n* = 511 autistic participants; Wechsler Abbreviated Scale of Intelligence, *n* = 777 autistic and 994 neurotypical participants; SSP hyposensitivity, *n* = 140 autistic and 82 neurotypical participants; SSP hypersensitivity, *n* = 137 autistic and 83 neurotypical participants; AQ, *n* = 181 autistic and 158 neurotypical participants; SRS-2, *n* = 508 autistic and 535 neurotypical participants. *P < 0.05; **P < 0.01; ***P < 0.001.

Finally, using support vector regression (SVR) with fivefold cross-validation, we tested whether FC deviations at each spatial scale predict clinical and cognitive measures in autistic individuals. At the connection level, FC deviations significantly predicted full-scale intelligence quotient (IQ; *r*_median _= 0.16; *P*_FDR_ <0.001) and Social Responsiveness Scale (SRS) scores (*r*_median _= 0.29; *P*_FDR_ <0.001). At the regional level, deviations predicted full-scale IQ (*r*_median _= 0.1; *P*_FDR_ = 0.01) and SRS (*r*_median _= 0.1; *P*_FDR_ = 0.02). At the network level, deviations predicted ADI social interaction (r_median _= 0.17; *P*_FDR_ <0.001), Autism Spectrum Quotient (AQ; *r*_median_ = 0.17; *P*_FDR_ <0.02) and SRS (*r*_median _= 0.18; *P*_FDR_ <0.001; Fig. 4). These results suggest that different levels of network organization capture distinct clinical features of autism.

## Discussion

Our work demonstrates that FC in autism exhibits scale-dependent heterogeneity, characterized by expected levels of variability at the level of individual connections but a significant convergence of atypical FC on specific brain regions and macroscopic networks. These findings indicate that autism is not uniformly heterogeneous across all levels of brain organization and provide a neurobiological framework for understanding both individual differences and shared clinical features in people meeting diagnostic criteria for the condition.

### Connection-level deviations are heterogeneous

The connection-level heterogeneity observed in people with autism was high, with no more than 4% of people showing an extreme deviation in the same connection. This result indicates that there is no single connection that is likely to play a major role in driving the core characteristics of the autism phenotype. This finding aligns with normative modeling studies of brain structure showing minimal deviation overlap at finer spatial resolutions^9,10^.

The heterogeneous distribution of FC deviations across the connectome may represent a neural correlate of the noted clinical heterogeneity of autism and provides a plausible explanation for the inconsistent FC findings reported in autism literature^3^. However, autistic people did not show an excess number of deviations and the level of deviation overlap at any given connection was not higher or lower than the overlap observed in controls. The number and concentration of deviations at any given connection were therefore within normative expectations. The absence of group differences in the ratio of positive to negative extreme deviations further indicates that autistic individuals show no evidence for predominantly hypo- or hyperconnectivity. This result is consistent with our previous work demonstrating that autistic individuals exhibit complex patterns of both increased and decreased FC, on average^4^.

### Heterogeneous FC deviations converge on common regions and networks

We observed considerably higher overlap of FC deviations in autism at the level of brain regions and canonical functional networks than at the level of specific connections (Fig. 3c,d). This result aligns with recent work indicating that although deviations of regional gray matter volume are located in highly heterogeneous areas, they aggregate within common circuits and networks across autistic individuals^10^. When taken with the connection-level findings, these regional and network-level results indicate that a key feature of atypical FC in autism is not the total number of extreme deviations but their preferential concentration on connections linked to specific brain regions and networks.

Negative FC deviations (that is, atypically reduced FC) generally showed greater regional and network-level overlap in autistic individuals in somatomotor, frontal and temporal regions. At the network level, autistic individuals also showed greater negative deviation overlap for connections linking the somatomotor system with the dorsal and ventral attentional systems, the medial temporal lobe and the DMN. Atypical sensory processing and motor coordination are well documented in autism literature^19^. This result aligns with past work^4,20^ showing reduced FC within sensorimotor areas and between sensorimotor areas and attentional systems. Weaker FC between these networks has correlated with social difficulties and restricted and repetitive behaviors in previous work^4^.

Autistic individuals also showed greater overlap for positive deviations (that is, higher FC than expected) within the DMN and between the default mode and other systems. Our observation of increased overlap of positive FC deviations in the DMN highlights the complex role of the DMN in the neural underpinnings of the behavioral phenotype in autism. The DMN is closely associated with self-referential thought and is most active during periods of rest and introspection^21^. The increased connectivity of the DMN with other brain regions may contribute to a more inward-focused cognitive experience and a reduced inclination to engage with the external environment. This heightened connectivity can potentially explain tendencies toward introspection and diminished social interaction, whereas atypical sensory processing aligns with the reduced coupling observed within sensory systems^22^.

Positive deviations additionally showed greater overlap for frontoparietal network FC. This result aligns with studies reporting hyperconnectivity in the frontoparietal or cognitive control network in autistic individuals^23,24^. It suggests that these deviations could be associated with the behavioral inflexibility often seen in autistic individuals, as this network plays a key role in cognitive control and task switching^23,24^. We found that positive deviations also occurred more frequently between subcortical and cortical brain areas in autistic individuals, in line with past research showing increased connectivity in these systems^4,5^.

Collectively, these findings suggest that a core neural phenotype of autism that is shared across a large fraction of autistic individuals involves the reduced FC of sensorimotor areas and the increased FC of transmodal areas on the level of regions and networks, consistent with reports of altered hierarchical function in autistic individuals^25^. Critically however, our findings provide several insights into the degree of interindividual variability that one can expect in the expression of these region- and network-specific atypicalities and their associated clinical correlates. For instance, within the sensorimotor system, autistic people showed up to 55% higher overlap, especially for connections linking the medial temporal lobe and the somatomotor network. They also showed up to 84.4% overlap for positive deviations of FC between the DMN, frontoparietal network and the rest of the brain. Our finding that heterogeneous connection-level deviations converge onto common regions and networks aligns with lesion network mapping studies showing that brain lesions causing neuropsychiatric symptoms, despite heterogeneous locations, map onto shared functional circuits when projected onto normative connectivity data^26,27^. Siddiqi and colleagues demonstrated this principle for depression, where both lesions and therapeutic stimulation sites converged on a common circuit^28,29^. Our results extend this framework to neurodevelopmental conditions. Specifically, we find that although no single connection reliably distinguishes autistic from neurotypical individuals, FC deviations preferentially aggregate within specific networks or attach to specific regions. These findings suggest that any clinical interventions should be tailored to address the specific clinical phenomena associated with an individual’s regional or network profile of deviations rather than relying on the one-size-fits all approach that is implied by classical comparisons of group means. To this end, it will be important to understand both common and divergent characteristics of autistic individuals in people who do and do not share specific FC (or other) phenotypes. Such an analysis will require very large samples to appropriately parse the heterogeneity of deviations with the relevant neural systems.

### Clinical and behavioral predictions

Despite the normative level of heterogeneity observed in autism at the connection level, FC deviations significantly predicted intellectual ability and social functioning, suggesting that they nonetheless may contribute to interindividual variations in the clinical phenotype. Regional deviation degree also significantly predicted intellectual ability, highlighting that intellectual ability is captured at both the fine-grained level of single connections and the coarser regional level.

Scores from the SRS were significantly predicted by deviation patterns at all levels, whereas the ADI social functioning scale was predicted by network-level FC deviations. Both scales capture different dimensions of social functioning. The SRS reflects broader social responsiveness, including social awareness, social cognition, social motivation and communication. By contrast, the ADI social functioning scale assesses specific aspects of social interaction related to autism, such as reciprocal social interaction and peer relationships^30^. Together these relationships suggest that FC deviations reflect social functioning at all scales of FC analysis.

The AQ scale was predicted solely by deviations at the level of networks. The AQ is a self- or parent-administered questionnaire designed to measure the broader phenotype of autistic traits, capturing social skills, communication, imagination, attention to detail and task-switching ability, which may suggest a need for clinical attention rather than for diagnostic purposes^31^. Variability in these broader autistic traits may therefore be linked to the global macro-organization of FC.

### Limitations and conclusions

Our analysis approach relies on the choice of a threshold for (1) defining extreme deviation at the connection level and (2) computing overlap at the region and network-levels. We showed that our general conclusions are robust to the specific choices made for these thresholds, but the threshold chosen will necessarily influence the exact levels of overlap observed across individuals.

Owing to poor scan coverage in a large portion of the participants, our analysis did not include the cerebellum, which is thought to play an important role in autism pathophysiology^32^. Our sample also had an imbalanced sex ratio between the autism and control groups, which aligns with the higher prevalence of autism in male individuals compared with female individuals^33^. Future research should prioritize including more autistic female individuals to better understand the unique characteristics and needs of this group.

We observed group differences in head motion, as quantified using mean FD, which may raise concerns about our findings, particularly those within somatomotor areas. However, we found no significant correlations between deviation scores and mean FD, suggesting that residual motion effects are unlikely to explain our findings.

Our ability to detect deviations is reduced at the extremes of the age distribution (below 8 and above 35 years), where sparser training data lead to increased predictive uncertainty. The findings are most robust within the 8–35-year range, where we have the most coverage in our training data. This range covers 93% of autistic individuals in our sample.

Our analysis only included individuals without intellectual disability (IQ ≥70). As a result, our findings may not generalize to autistic individuals with co-occurring intellectual disability.

Our primary analysis focused on a threshold of *Z* = |2.3| for defining extreme deviations. This choice is somewhat arbitrary. Our supplementary analyses indicated that our main findings are robust to the use of both more lenient and more stringent thresholds (Supplementary Section 9). Future work may explore alternative, threshold-free methods for quantifying deviation heterogeneity across people.

The absence of race and ethnicity data limits our ability to assess the representativeness and generalizability of findings across racial and ethnic groups.

In summary, our results highlight the importance of adopting a multiscale approach to characterizing the heterogeneity of neural phenotypes in autism. This multiscale perspective reveals a novel organizational principle: although deviations at the level of specific connections are highly idiosyncratic, they converge into more consistent patterns at regional and network levels, offering a parsimonious account of how a common diagnosis might arise despite pronounced individual differences in underlying connectivity. Connection-level heterogeneity offers a plausible neural substrate for individual phenotypic differences and may explain the inconsistent FC findings reported in literature thus far. Our findings further suggest that the reduced FC of sensorimotor systems and increased FC of transmodal association networks potentially reflect imbalanced signaling along the sensorimotor-association axis of the brain. FC deviations at distinct levels predict different clinical phenotypes, emphasizing the importance of considering multiple levels when characterizing brain–behavior relationships. These results replicate across datasets and are robust across different granularities of brain parcellations and multiple sensitivity analyses.

## Methods

### Participants

We pooled scans from three large datasets: the European Autism Interventions (EU-AIMS) Longitudinal European Autism Project (LEAP)^34^ (https://www.eu-aims.eu/ and https://www.aims-2-trials.eu/) and the Autism Brain Imaging Data Exchanges I and II, or ABIDE 1 and ABIDE 2^5^, details can be found in Supplementary Section 1. Quality control and exclusion criteria are detailed in the Supplementary Section 2 and follow previous work^4^.

For the EU-AIMS LEAP cohort, written informed consent was obtained from all participants or their legal guardians before participation, in accordance with the protocols approved by the local ethics committees at each participating site^34^. For the ABIDE 1 and 2 datasets, all data were collected under protocols approved by the local institutional review boards of each contributing site, and informed consent was obtained from all participants or their legal guardians^5,35^.

Data collected within the ABIDE 1 and 2 initiatives are available for public use on the following links: http://fcon_1000.projects.nitrc.org/indi/abide/abide_I.html; and http://fcon_1000.projects.nitrc.org/indi/abide/abide_II.html.

Data collected in LEAP are stored and curated at the central EU-AIMS database at the Pasteur Institute in Paris. LEAP data are accessible to consortium members who submit an analysis proposal, and it is available for use to the wider research public via a secure database (https://elixir-luxembourg.org/).

The final sample included 796 autistic individuals (141 female participants; age range 5–58 years) and 1,028 neurotypical individuals (256 female participants; age range 5–56 years) recruited across 32 different sites. Table 1 contains detailed information on the clinical and demographic characteristics of the participants included in the study.

**Table 1:** Demographic and clinical information

	EU-AIMS LEAP			ABIDE 1			ABIDE 2			All		
	Autism	NT	*t* value, *P* value	Autism	NT	*t* value, *P* value	Autism	NT	*t*-Value, *P* value	Autism	NT	*t*-value, *P* value
*n*	222	200	–	369	481	–	205	347	–	796	1028	–
Male/female^a^	158/64	131/69	1.31, 0.251	321/48	390/91	**4.9, 0.026**	176/29	251/96	**12.68, 0.0003**	655/141	772/256	**13.2, 0.0002**
Age, years (mean ± s.d.)	18.1 ± 5.7	18.3 ± 5.8	−0.4, 0.7	17.0 ± 7.8	17.1 ± 7.3	−0.3, 0.8	14.0 ± 7.0	13.0 ± 5.7423	1.931, 0 .0539	16.2 ± 7.0	15.95 ± 6.9	1.7, 0.1
Full-scale IQ (mean ± s.d., *n*)	105.5 ± 15.2, 221	108.5 ± 12.6, 199	**−2.2, 0.028**	106.4 ± 16.3, 361	111.4 ± 12.4, 464	**−5.0**, <**0.0001**	106.1 ± 15.7, 195	115.4 ± 12.7, 331	**−7.4**, <**0.0001**	106.1 ± 15.8, 777	112.2 ± 12.8, 994	**−8.97**, <**0.0001**
Head motion mean FD (mean ± s.d.)	0.076 ± 0.043	0.069 ± 0.035	1.933, 0.054	0.081 ± 0.034	0.071 ± 0.031	**4.41**, <**0.0001**	0.077 ± 0.035	0.075 ± 0.033	0.9, 0.365	0.079 ± 0.037	0.072 ± 0.033	**4.18**, <**0.0001**
Handedness (right/left/ambidextrous, *n*)	158/26/6, 190	144/15/4, 163	–	207/30/6, 243	300/25/6, 331	–	158/17/19, 194	310/16/14, 340	–	523/73/31, 627	754/56/24, 834	–
Current medication use	84	12	–	85	2		38	7	–	207	21	–
ADOS social affect (mean ± s.d., *n*)	5.7 ± 2.5, 217	–	–	8.8 ± 4.2, 189	–	–	9.3 ± 3.7, 105	–	–	7.6 ± 3.9, 509	–	–
ADOS RRB (mean ± s.d., *n*)	4.5 ±2.6, 217			2.7 ± 1.8, 187			3.0 ±1.8, 105			3.5 ± 2.3, 511		
ADI social (mean ± s.d., *n*)	15.7 ± 6.6, 212	–	–	19.8 ± 5.3, 255	–	–	18.4 ± 5.3, 117	–	–	18.0 ± 6.1, 584	–	–
ADI communication (mean ± s.d., *n*)	12.9 ± 5.6, 212	–	–	15.9 ± 4.5, 256	–	–	14.8 ± 4.5, 116	–	–	14.6 ± 5.1, 584	–	–
ADI RRB (mean ± s.d., *n*)	3.9 ± 2.5, 212	–	–	6.0 ± 2.5, 256	–	–	5.3 ± 2.3, 117	–	–	5.1 ± 2.6, 585	–	–
SSP Hypo. (mean ± s.d., *n*)	31.4 ± 6.9, 140	37.6 ± 3.6, 82	**−7.4,** <**0.0001**	–	–	–	–	–	–	31.4 ± 6.9, 140	37.6 ± 3.6, 82	**−7.4,** <**0.0001**
SSP Hyper. (mean ± s.d., *n*)	54.3 ± 10.4, 137	67.4 ± 3.9, 83	**−10.9,** <**0.0001**	–	–	–	–	–		54.3 ± 10.4, 137	67.4 ± 3.9, 83	**−10.9,** <**0.0001**
SRS (mean ± s.d., *n*)	85.2 ± 30.2, 178	19.9 ± 14.3, 93	**19.74**, <**0.0001**	91.0 ± 30.3, 160	21.9 ± 16.8, 165	**25.5**, < **0.0001**	86.6 ± 30.2, 170	20.0 ± 15.0, 277	**31.0**, <**0.0001**	87.5 ± 30.3, 508	20.6 ± 15.4, 535	**45.33**, <**0.0001**
AQ (mean ± s.d., *n*)	89.7 ± 20.6, 181	43.8 ± 16, 158	**22.6**, <**0.0001**	–	–	–	–	–	–	89.7 ± 20.6, 181	43.8 ± 16, 158	**22.6**, <**0.0001**
Ethnicity White/Black/Asian/mixed/other	163/1/1/17/3	148/1/4/10	–	–	–	–	–	–	–	163/1/1/17/3	148/1/4/10	–

Two-sample *t*-tests (two-sided, uncorrected for multiple comparisons) were performed to test group differences for each variable, except for sex (see ^a^). NT, neurotypicals; ‘–’, not applicable. A bold statistic indicates a significant difference between groups.

^a^Chi-squared test was performed to test the sex ratio difference between groups.

### Race and ethnicity

In the EU-AIMS LEAP cohort, self-identified race was collected via parental- or self-report and categorized as white, Asian, Black, mixed or other, following the classification system adopted by the participating clinical sites^36^; numbers can be found in Table 1. For the ABIDE 1 and 2 datasets, race or ethnicity was not collected as part of the shared phenotypic protocol. ABIDE is a retrospective, multisite data bank aggregated from previously and independently collected datasets drawn from multiple international sites across North America and Europe^5,35^. Demographic harmonization was limited to variables consistently available across all contributing sites (that is, age, sex, handedness and IQ), and race/ethnicity was not among them.

### Clinical diagnosis

Autistic participants in EU-AIMS LEAP met DSM-IV/5 or ICD-10 criteria, with most confirmed by Autism Diagnostic Interview-Revised (ADI-R)^30^ and/or Autism Diagnostic Observation Schedule (ADOS)-2^37^ (see ref. ^36^ for more details). ABIDE sites used varying diagnostic procedures, though most used ADOS^38^ and/or ADI-R^30^. Control participants had no psychiatric diagnoses. See Supplementary Section 8 for more details.

### MRI acquisition and preprocessing

Resting-state fMRI and structural scans were obtained using 3T magnetic resonance imaging (MRI) scanners at 32 scanning sites and preprocessed with rigorous quality control, as per prior work (see Supplementary Sections 3 and 4 for details).

### Mapping FC

We mapped the interregional FC using the Schaefer parcellation^16^ with 400 cortical regions of interest and 15 subcortical regions of interest from the Harvard–Oxford atlas^17^. We performed additional analyses with Schaefer-200 and Schaefer-800 parcellations. After excluding 25 regions with low coverage (<70% coverage in >5% participants), 390 regions remained (Supplementary Fig. 1). Schaefer regions were assigned to seven networks^18^, with subcortical regions labeled as thalamus, striatum or medial temporal lobe. FC matrices were computed using Pearson correlations and normalized via Gaussian-gamma mixture modeling for enhanced differentiation of signal from noise^39^. This approach separates meaningful connectivity values from background noise by modeling their distinct statistical distributions, effectively suppressing connections likely to be noise. ComBat^40^ was used to remove scan-site effects. See Supplementary Section 5 for further details on the Gaussian-gamma mixture modeling thresholding.

### Normative modeling

We applied Gaussian process regression to fit normative models predicting FC for each of 75,855 pairs of brain regions using age, sex and mean FD (an aggregate measure of head motion; Supplementary Section 4). For each connection in each person, we quantified deviations from normative model expectations using *z*-scores, calculated by subtracting predicted from observed FC values, divided by estimated variance. To obtain deviations for the group of autistic individuals, the model was trained on neurotypical and tested on autistic participants, establishing their deviations from the normative model. To obtain deviations for neurotypical individuals, we used tenfold cross-validation, where we trained the model on nine folds and tested it on the tenth held-out fold. This was repeated across all folds to obtain deviation estimates for the entire sample, whereby each control participant’s deviations were computed from a model trained without their data. Separately, to assess whether the normative modeling procedure was successful, we computed normative model validation statistics, which can be found in Supplementary Section 7. Our primary outcomes were individual-level FC deviations from normative expectations, quantified as *z*-scores, examined at three spatial scales: pairwise connections, brain regions and canonical functional networks.

### Connection-level analysis

Extreme deviations were defined as |*Z*| > 2.3, corresponding to approximately *P* < 0.01. Thresholds for defining extreme deviations in normative modeling studies have typically ranged from |*Z*| > 1.96 (*P* < 0.05)^41,42^ to |*Z*| > 2.6 (*P* < 0.005)^11,12,43^. The present threshold represents a principled intermediate choice that balances sensitivity to meaningful deviations against specificity. Given that a central aim of this study is to characterize heterogeneity in autism and capture the diversity of individual-level atypical connectivity patterns, we favored a threshold that maintains sensitivity rather than a more stringent cutoff that risks obscuring genuine variability. To ensure robustness, we report supplementary analyses at |*Z*| > 1.96 (*P* < 0.05), |*Z*| > 2.6 (*P* < 0.005) and |*Z*| > 3.1 (*P* < 0.001), which demonstrate consistent spatial patterns with the expected attenuation at more extreme thresholds (Supplementary Section 9 and Supplementary Figs. 5 and 6). Group differences in total extreme deviations were assessed using Wilcoxon rank-sum tests for both positive and negative deviations. We compared positive-to-negative deviation ratios between groups using independent two-sample *t*-tests to assess polarity bias in autistic versus neurotypical individuals. Interindividual heterogeneity was analyzed by calculating the percentage of participants showing extreme deviations per connection, with significance determined through 10,000 group-label permutations and FDR_BH_ correction (Fig. 2a).

### Region-level analysis

For each participant, we counted the number of connections with extreme *z*-scores attached to each brain region (|*z*| > 2.3; Fig. 2b), a quantity we term deviation degree. We then used deviation degree to study the level of deviation overlap at the regional level. At the connection level, each FC deviation estimate can be classified as deviant or not depending on whether it exceeds the threshold. Computing overlaps across participants in this scenario is straightforward. However, at the region level, deviation degree is not binary (for example, a region might have 0, 3 or 12 deviant connections), complicating attempts to quantify overlap across participants. One approach would be to apply a threshold to deviation degree values, but the specific threshold value that should be used is unclear. We therefore evaluated group differences across a range of thresholds, 1 < *τ* < 20, and, at each threshold, plotted how many participants had a deviation degree of at least *τ* (Fig. 3b). The upper bound of 20 was chosen because few people showed higher deviation degree within any brain region. We then calculated the AUC across these thresholds, providing a single summary measure that captures regional overlap across groups without depending on any particular threshold choice. We compared AUC values between autistic and control groups using 10,000 permutations of diagnostic labels and used false discovery rate correction across all regions.

### Network-level analysis

We used the same approach to examine group differences at the network level, calculating the percentage of extreme deviations that occurred within and between brain networks (Fig. 2c). For each network and group, we calculated participant overlap across deviation thresholds (1–10%), with the upper bound of 10% chosen because very few participants showed deviation overlap beyond this threshold (Fig. 3b). We compared these patterns between groups. The inference was performed with 10,000 permutations and FDR_BH_-corrected *P* values.

### Predicting clinical and cognitive variables from FC deviations

As secondary outcomes, we assessed whether deviation patterns predicted clinical and cognitive measures. We used SVR to develop multivariate predictive models of clinical and cognitive measures using FC deviation scores at the connection, region and network levels. Models were fitted to the following measures: ADOS social affect, ADOS restricted interests and repetitive behavior (RRB), ADI RRB, ADI communication, ADI social interaction, SRS-2, full-scale IQ from the Wechsler Abbreviated Scales of Intelligence Second Edition and the Short Sensory Profile (SSP) scale and AQ^31^. See Table 1 and Supplementary Section 8 for descriptive statistics and more information on the variables. See Supplementary Section 11 for model details.

### Reporting summary

Further information on research design is available in the Nature Portfolio Reporting Summary linked to this article.

## Supplementary information

Supplementary Information Supplementary Sections 1–12, with Figs. 1–10 and Tables 1–5.

Reporting summary

Peer Review File

## Data Availability

Data collected within the ABIDE 1 and 2 initiatives are available for public use on the following links: http://fcon_1000.projects.nitrc.org/indi/abide/abide_I.html and http://fcon_1000.projects.nitrc.org/indi/abide/abide_II.html. Data from the EU-AIMS LEAP consortium are stored at the central EU-AIMS database at the Pasteur Institute in Paris. These data are currently only accessible to consortium members with an analysis proposal approved and will become publicly available via a secure database in the near future (https://elixir-luxembourg.org/).

## Code availability

Analysis code used for this manuscript is available via GitHub at https://github.com/ivaili/MultiscaleHeterogeneity. Software versions for the data analysis are: FMRIB Software Library 5.0.10, Matlab R2018b, Spyder(Python 3.12) and Predictive Clinical Neuroscience Toolkit 0.20, and scikit-learn 0.24.2 code for the analyses is available via GitHub at https://github.com/ivaili/MultiscaleHeterogeneity.
