# Supplementary material for: Multiscale heterogeneity of atypical functional connectivity in autism
Source: Nat Ment Health. 2026 Jun 1;4(6):1010–20. doi: 10.1038/s44220-026-00656-y (PMC13259941; doi:10.1038/s44220-026-00656-y)
Supplement: Supplementary file 1 — Supplementary Sections 1–12, with Figs. 1–10 and Tables 1–5. [file 44220_2026_656_MOESM1_ESM.pdf]

nature mental health

Supplementary information https://doi.org/10.1038/s44220-026-00656-y

Multiscale heterogeneity of atypical functional connectivity in autism

In the format provided by the authors and unedited

Supplementary information
Multiscale heterogeneity of atypical functional connectivity in autism
Ilioska et al.

Section 1. Data 2
Section 2. Exclusion Criteria 2
Figure S1. A diagram of available and excluded participants during data quality control 4
Section 3. MRI Acquisition and Pre-processing..4
Figure S2. ROIs excluded due to low coverage 6
Section 4. Evaluation of Motion-Artifact Removal..7
Figure S3. QC-FC distributions and distance dependence..8
Table S1. Scanning parameters for each scanning site in the LEAP consortium 9
Table S2. Scanning parameters for each scanning site in the ABIDE 1 cohort....................................10
Table S3. Scanning parameters for each scanning site in the ABIDE 2 cohort....................................11
Section 5. Application of Mixture Modeling Normalization..11
Section 6. Removal of scanning-site-specific variance 12
Figure S4. Evaluation of scanning site effects before and after ComBat harmonization. 13
Section 7. Normative modeling evaluation 13
Figure S5. Distributions of the normative modelling evaluation metrics for the group of neurotypical individuals. 13
Section 8. Behavioral variables 13
Section 9. Results for different thresholds and parcellations 14
Figure S6. Overlap of negative and positive functional connectivity (FC) deviations at the level of regions, and networks for alternative thresholds. 15
Figure S7. Threshold sensitivity analysis at |Z| > 2.6. 16
Figure S8. Parcellation resolution sensitivity analysis...16
Section 10. Sensitivity analyses..17
Table S4. Pearson correlations of sensitivity analyses results with the original analysis result....................................17
Section 11. Brain- behavior relationship 17
Section 12. Dataset Sensitivity and stability Analysis..17
Table S5. Sensitivity to dataset 18
Figure S9. Bootstrap reliability analysis of network- and region-level deviation overlap. 19
Figure S10. Age distribution of the group of neurotypical individuals 20

Section 1. Data
We used data from three consortia: the EU-AIMS Longitudinal European Autism Project (LEAP), accessible at (https://www.eu-aims.eu/ and https://www.aims-2-trials.eu/), and the Autism Brain Imaging Data Exchange (ABIDE) 1 and ABIDE 2, available at (http://fcon_1000.projects.nitrc.org/indi/abide/).

The LEAP consortium focuses on the identification and classification of autism biomarkers across multiple research centers [1]. A total of 437 autistic and 300 neurotypical individuals were recruited across five sites. Male and female participants, aged between 6 and 30 years, underwent standardized MRI protocols and comprehensive clinical, cognitive, and genetic assessments. For a more in-depth understanding of the LEAP study, refer to [1,2].

ABIDE is an open access initiative, providing access to collected rs-fMRI and structural MRI data from autistic participants and matched controls [3,4]. Individual studies were initially conducted at various sites before being combined into a single dataset. ABIDE 2 expands upon the ABIDE 1 initiative, and collectively, these efforts offer 2,226 unique datasets from 32 sites, including data from 1,060 autistic individuals and 1,166 controls aged between 5 and 64.

Section 2. Exclusion Criteria

Of the 2,635 participants with available demographic and resting-state fMRI data across the three datasets (LEAP: 623; ABIDE I: 1,110; ABIDE II: 902), 811 (30.8%) were excluded through sequential quality control steps. Exclusions were made for preprocessing failure (N=25), incomplete scans (N=225), excessive head motion (N=426; mFD > 0.25mm, >20% of framewise displacements above 0.2mm, or any single displacement > 5mm), poor signal coverage (N=20), visible artifacts identified through carpet plot inspection (N=36), IQ below 70 (N=75), and structural brain abnormalities (N=4). The final sample comprised 1,824 participants (LEAP: 422; ABIDE I: 850; ABIDE II: 552), including 796 autistic individuals (141 female) and 1,028 neurotypical individuals (256 female), aged 5–58 years, recruited across 32 sites.

Initially, the LEAP dataset contained 623 participants with available demographic and rs-fMRI data. Subjects with low-quality resting-state fMRI scans due to preprocessing failure (N=2) and those with incomplete scans (N=8) were excluded. To account for head motion contamination during the resting-state fMRI scan, participants were excluded if they met at least one of the following criteria: a mean framewise-displacement (mFD) > 0.25mm, more than 20% of FDs above 0.2mm, or any FDs larger than 5mm (N=123). These criteria are consistent with the "stringent" exclusion guidelines reported in previous research [5,6].

We next excluded parcellated ROIs with over 30% signal dropout in more than 5% of the entire subject sample (Figure S2) and subjects who had poor coverage (<70%) in more than 10% of the remaining regions. In the LEAP cohort, this procedure did not result in any exclusions (N=0).

Carpet plots for each participant were then visually inspected [7,8]. Displaying the complete fMRI time series in matrix form with a normalized color scale representing signal fluctuations at each time point in each voxel, these plots proved effective in identifying significant imaging artifacts and signal contributions from brain-wide signal changes. An additional 17 participants with noticeable artifacts were excluded based on these plots. All participants with an IQ < 70 (N=51) were excluded, resulting in a final total of 422 subjects for analysis.

In the ABIDE 1 cohort, demographic and resting-state fMRI data were available for 1,110 participants. Participants with low-quality scans due to preprocessing failure (N=23) and those with incomplete scans (N=8) were excluded. To account for excessive head motion during the resting-state fMRI scan, we applied the same criteria as in the LEAP dataset resulting in N=173 exclusions. A total of 20 participants were excluded for poor signal/anatomical coverage. Participants with visible carpet-plot-artifacts (N=10) were removed. All participants

with IQ < 70 (N=22) and those with structural brain abnormalities (N=4) were also excluded, resulting in a final count of 850 subjects included in the analysis.

For the ABIDE 2 cohort, data from sites with TR > 800ms were considered, and demographic and resting-state fMRI data were available for 902 participants. We excluded participants with incomplete scans (N=209) and excessive head motion (N=130). No participants were excluded based on low coverage (N=0). Following visual inspection of functional data carpet plots [7], participants with visible artifacts (N=9) were excluded. All participants with IQ < 70 (N=2) were also excluded, resulting in a final total of 552 subjects for analysis. Total exclusion for each step are shown on Figure S1.

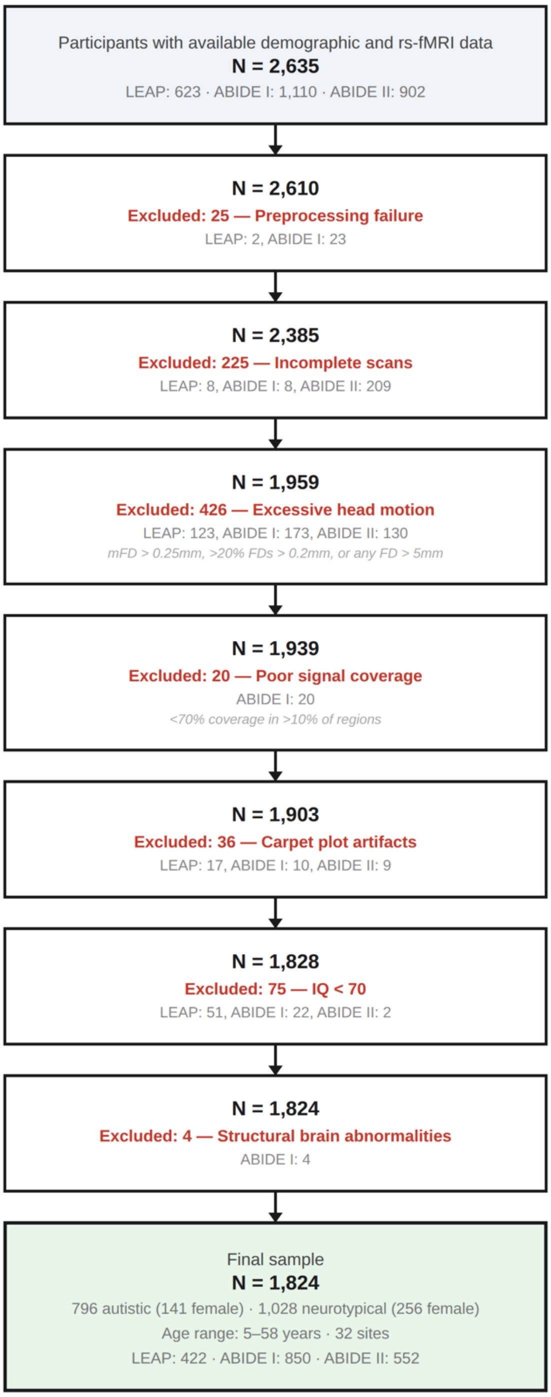

Figure S1. A diagram of available and excluded participants during data quality control

Section 3. MRI Acquisition and Pre-processing

MRI data in the LEAP consortium were collected at five locations: Cambridge University, King's College London (KCL), Central Institute of Mental Health Mannheim, Radboud University Nijmegen Medical Centre, and University Medical Center Utrecht, using a uniform MRI protocol. A magnetization prepared rapid gradient-echo (MPRAGE) sequence, following the ADNI 2 / GO protocol (http://adni.loni.usc.edu), was used

for structural scans, and a multi-echo planar imaging sequence was employed for resting-state fMRI scans [9]. Table S1 provides further information on scanning acquisition parameters for the LEAP cohort.

For the ABIDE 1 cohort, data from sixteen sites were analyzed, including: California Institute of Technology (Caltech), Carnegie Melon University (CMU), Kennedy Krieger Institute (KKI), University of Leuven (Leuven), Ludwig Maximilian's University Munich (MaxMun), NYU Langone Medical Center (NYU), Olin Institute of Living at Hartford Hospital (Olin), University of Pittsburgh School of Medicine (Pitt), Social Brain Lab BCN Neuroimaging Center, University Medical Center Groningen (SBL), San Diego State University (SDSU), Stanford University (Stanford), Trinity Centre for Health Sciences (Trinity), University of California Los Angeles (UCLA), University of Michigan (UM), University of Utah School of Medicine (USM), and Yale School of Medicine,Yale Child Study Center (Yale). Table S2 offers a detailed overview of acquisition parameters for each ABIDE 1 site.

Thirteen sites contributed data to the ABIDE 2 cohort analysis: Erasmus University Medical Center (EMC), Rotterdam, ETH Zürich (ETH), Georgetown University (GU), Indiana University (IU), Kennedy Krieger Institute (KKI), Katholieke Universiteit Leuven (KUL), NYU Langone Medical Center Sample 1 (NYU1), NYU Langone Medical Center Sample 2 (NYU2), San Diego State University (SDSU), Stanford University (Stanford), Trinity Centre for Health Sciences (TCD), University of California David (UCD), University of Utah School of Medicine (USM), and University of Miami (Miami). Table S3 provides further details on acquisition parameters for each ABIDE 2 sites.

All rs-fMRI images underwent preprocessing using the FMRIB Software Library (FSL; www.fmrib.ox.ac.uk/fsl) [10]. The following pipeline was implemented: removal of the initial five volumes for signal equilibration, volume realignment to the median volume using MCFLIRT for primary head motion correction, grand mean scaling, and spatial smoothing employing a 6mm FWHM Gaussian kernel. ICA-AROMA was then used for secondary head motion-related artifact correction [11]. ICA-AROMA is capable of effectively removing motion-related artifacts while preserving neurobiological signals of interest [12], and has compares favorably to alternative methods for motion-related confound removal [5]. Mean signals from the CSF and white matter were regressed out as nuisance covariates, and a 0.01Hz temporal high-pass filter was applied. Participant functional images were co-registered to their respective anatomical images using boundary-based registration in FSL FLIRT [13,14]. High-resolution structural images were registered to MNI152 standard space using a 12-parameter affine transformation and further refined with a non-linear registration via FSL FNIRT, employing a 10mm warp and 2mm resampling resolution [10]. Finally, functional images were normalized to 2mm MNI152 standard space by applying the transformation of the functional image to T1 and T1 to MNI152. All subsequent analyses were conducted in MNI152 standard space.

We additionally excluded a further 57 participants from sites with very few (i.e., <10) female participants: the University of Leuven sample 2; Social Brain Lab, UMC Groningen; and the Erasmus Medical Center Rotterdam.

The final sample included 796 autistic individuals (141 females; age-range: 5-58) and 1028 NT individuals (256 females; age-range: 5-56).

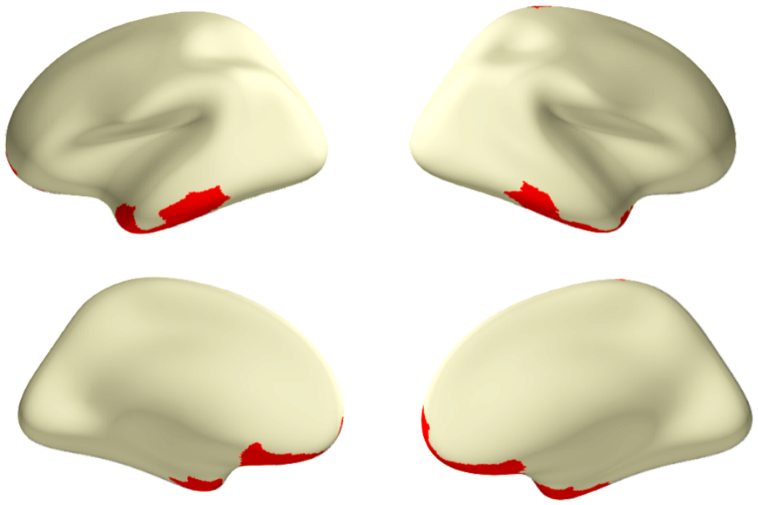

Figure S2. ROIs excluded due to low coverage
ROIs were omitted (red) when they exhibited low coverage (less than 70% of the voxels within the ROI) in over 5% of the participants.

Section 4. Evaluation of Motion-Artifact Removal

The quality control-functional connectivity (QC-FC) correlation denotes the association between the mean framewise displacement and functional connectivity for each edge. More specifically, it represents the correlation between observed FC values across participants and the participants' mean framewise displacement at each edge. This measure offers insights into the dependency of the FC value on head motion, reflecting the quality of the FC data. To estimate this relationship post-denoising, we computed the QC-FC correlations [5,15].

In-scanner head motion is thought to bias connectivity estimates between two nodes in relation to the distance between the nodes [15-17]. This observation is particularly relevant for neurodevelopmental cohorts, where increased in-scanner motion distinguishes patients from controls [17]. Subject movement may enhance short-distance connections while diminishing long-distance connections [15-17]. To assess the residual distance dependence of subject movement post-denoising for two pipelines, we calculated the QC-FC distance dependence [5]. We initially employed the centre of mass for each node to determine the Euclidean distance between the nodes i and j. Subsequently, we computed the correlation between the distance separating each node pair and the QC-FC correlation of the edge linking those nodes; this correlation served as an estimate of the distance-dependence of motion artifacts.

Figure S3 displays the QC-FC distributions for our primary and alternative pipelines, as well as distance dependence for evaluating residual motion contamination in our sample. All QC-FC values fell within the range of $-0.3 < r < 0.3$, with 95% of the values situated between $-0.15 < r < 0.16$, signifying that residual correlations between FC and head motion were minimal. These values are comparable to previous benchmarking studies on pipeline denoising efficacy, where some of the most effective denoising pipelines exhibited QC-FC values ranging between $-0.45 < r < 0.45$ [5]. Similarly, although some QC-FC distance dependence persists, the relationship aligns with earlier findings [5], underscoring the relative success of our denoising methodology.

Additionally, we tested the correlation between mean FD and the deviation scores (Z-scores) for every connection in both groups. No significant correlations were observed after correction for multiple comparisons, even when partialling the effects of age and sex, indicating that residual motion effects do not systematically driving the deviation scores.

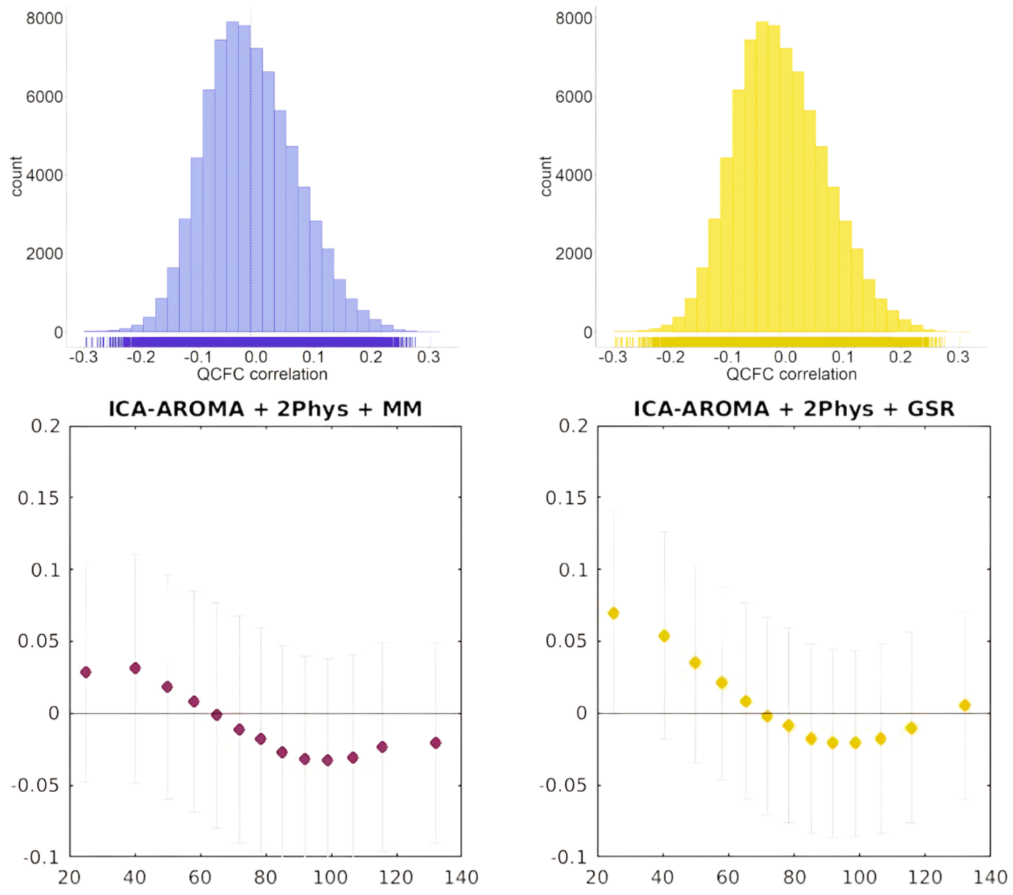

Figure S3. QC-FC distributions and distance dependence
The distributions of QC-FC correlations across edges indicate that this dependence is minimal, with both preprocessing pipelines yielding distributions centred near zero. The QC-FC distance dependence plots show the relationship between QC-FC correlation and the spatial distance of each edge. For every bin, the mean QC-FC for the ROI distance bin (circle) and the standard deviation (error bar) of the QC-FC correlation are presented.

Table S1. Scanning parameters for each scanning site in the LEAP consortium

				EPI BOLD multi-echo imaging sequence			
**Site**	Scanner	Field str.	Instruct.	TR /TE1/TE2 / TE3 (ms)/FA (°)	No. of vol.	No. of slices	Voxel size
**Cambridge**	Siemens Magnetom Verio	3 T	Fixation	2300/ 12/29/46/80	266	33	3.8 x 3.8 x 3.8
**KCL**	GE NA	3 T	Fixation	2300/ NA/31/48/90	215	33	3.8 x 3.8 x 3.8
**Mannheim**	Siemens Magnetom TIM Trio	3 T	Fixation	2300/ 12/29/46/80	215	33	3.8 x 3.8 x 3.8
**Nijmegen**	Siemens Magnetom Skyra	3 T	Fixation	2300/ 12/NA/NA/80	266	33	3.8 x 3.8 x 3.8
**Utrecht**	Philips	3 T	Fixation	2300/ 13/31/49/80	200	33	3.75 x 3.75 x 3.75

Table S2. Scanning parameters for each scanning site in the ABIDE 1 cohort

				EPI BOLD imaging sequence			
**Site**	Scanner	Field str.	Instruct.	TR /TE (ms)/FA (°)	No. of vol.	No. of slices	Voxel size
**Caltech**	Siemens Magnetom Verio	3 T	Eyes closed	2000/30/75	150	34	3.5 x 3.5 x 3.5
**CMU**	Siemens Magnetom Verio	3 T	Eyes closed	2000/30/73	240	28	3.0 x 3.0 x 3.0
**KKI**	Philips Achieva	3 T	Fixation	2500/30/75	156	47	3.0 x 3.0 x 3.0
**Leuven**	Philips	3 T	Fixation	1667/33/90	250	32	3.6 x 3.6 x 4.0
**MaxMun**	Siemens Magnetom Verio	3 T	Fixation	3000/30/80	120/200	40	3.0 x 3.0 x 3.0
**NYU**	Siemens Magnetom Allegra	3 T	Fixation	2000/15/90	180	33	3.0 x 3.0 x 4.0
**Olin**	Siemens Magnetom Allegra	3 T	Fixation	1500/27/60	210	29	3.4 x 3.4 x 4.0
**Pitt**	Siemens Magnetom Allegra	3 T	Eyes closed	1500/25/70	200	29	3.1 x 3.1 x 4.0
**SBL**	Philips Intera	3 T	Eyes closed	2200/30/80	200	38	2.75 x 2.75 x 2.72
**SDSU**	GE MR750	3 T	Fixation	2000/30/90	180	41	3.4 x 3.4 x 3.4
**Stanford**	GE SIGNA	3 T	Eyes closed	2000/30/80	180	29	3.1 x 3.1 x 4.5
**Trinity**	Philips Achieva	3 T	Eyes closed	2000/28/90	150	38	3.0 x 3.0 x 3.5
**UCLA**	Siemens Magnetom TIM Trio	3 T	Fixation	3000/28/90	120	34	3.0 x 3.0 x 4.0
**UM**	GE SIGNA	3 T	Fixation	2000/30/90	300	40	3.44 x 3.44 x 3.0
**USM**	Siemens Magnetom TIM Trio	3 T	Eyes open	2000/28/90	240	40	3.4 x 3.4 x 3.0
**Yale**	Siemens Magnetom TIM Trio	3 T	Eyes open	2000/25/60	200	34	3.4 x 3.4 x 4.0

Table S3. Scanning parameters for each scanning site in the ABIDE 2 cohort

				EPI BOLD imaging sequence			
**Site**	Scanner	Field str.	Instruct.	TR /TE (ms)/FA (°)	No. of vol.	No. of slices	Voxel size
**EMC**	GE MR750	3 T	Eyes closed	2000/30/85	160	37	3.6 x 3.6 x 4.0
**ETH**	Philips Achieva	3 T	Fixation	2000/25/90	210	40	3.0 x 3.1 x 3.0
**GU**	Siemens Magnetom TIM Trio	3 T	Eyes open	2000/30/90	154	43	3.0 x 3.0 x 2.5
**IU**	Siemens Magnetom TIM Trio	3 T	Eyes open	813/28/60	433	42	3.4 x 3.4 x 3.4
**KKI**	Philips Achieva	3 T	Fixation	2500/30/75	156	47	3.0 x 3.0 x 3.0
**KUL**	Philips	3 T	Fixation	2500/30/90	162	45	2.5 x 2.5 x 2.7
**NYU1**	Siemens Magnetom Allegra	3 T	Fixation	2000/15/90	180	33	3.0 x 3.0 x 4.0
**NYU2**	Siemens Magnetom Allegra	3 T	Fixation	2000/30/82	180	34	3.0 x 3.0 x 3.0
**SDSU**	GE MR750	3 T	Fixation	2000/30/90	180	41	3.4 x 3.4 x 3.4
**Stanford**	GE SIGNA	3 T	Eyes closed	2000/30/80	180	31	3.4 x 3.4 x 3.5
**TCD**	Philips Achieva	3 T	Fixation	2000/27/90	210	37	3.0 x 3.0 x 3.2
**UCD**	Siemens Magnetom TIM Trio	3 T	Eyes open	2000/24/90	460	36	3.5 x 3.5 x 4.0
**USM**	Siemens Magnetom TIM Trio	3 T	Eyes open	2000/28/90	240	40	3.4 x 3.4 x 3.0
**Miami**	GE Healthcare	3 T	Eyes closed	2000/30/75	290	NA	3.4 x 3.4 x 3.0

Section 5. Application of Mixture Modeling Normalization

Mixture modeling normalization offers a statistically principled approach for addressing a fundamental challenge of functional connectivity studies: namely, that the precise threshold that should be used to distinguish meaningful from spurious correlation values is often unclear. The method works by decomposing the distribution of connectivity values into signal and noise components. After applying a Fisher-z transformation to the correlation values, a three-component mixture model is fitted: a central Gaussian distribution captures the bulk of near-zero values (interpreted as noise), while two Gamma distributions capture the positive and negative tails (interpreted as genuine signal). The Gamma distributions are thus used to capture the meaningful FC estimates (both positive or negative), which are relatively sparse and appear in the tails of the distribution of FC values.

In a second step, each connectivity value across all three distributions is z-score normalized using the mean and standard deviation of the noise distribution. The resulting z-values quantify, in units of standard deviation, the degree to which a given FC estimate deviates from the noise floor. Connections hovering near zero remain near zero, while those representing true signal are amplified in proportion to their deviation from noise. In this way,

the procedure acts as a soft threshold that enhances signal-to-noise ratio without imposing a hard cutoff that arbitrarily discards potentially meaningful connections. For further details, see [18-24].

Section 6. Removal of scanning-site-specific variance

To mitigate the impact of scanning site variations, we employed ComBat [25], a harmonization method that uses multivariate linear mixed-effects regression coupled with empirical Bayes. This method is well-documented for its effectiveness in removing site-related artefacts in multi-site resting-state fMRI studies [26]. In our FC analysis, ComBat was applied while preserving the variance attributed to diagnostic group, age, and sex, while effectively normalizing differences across sites. Figure S4 demonstrates the effectiveness of the ComBat harmonization procedure on our data. Prior to harmonization, most FC estimates showed significant site effects ($p < 0.05$) and a linear SVM classifier could readily distinguish scanning sites from the connectivity data, with AUC ROC scores well above chance for nearly all site pairs. After ComBat, significant site effects were virtually eliminated across all connections, and classification performance dropped to near chance levels, confirming that site-related variance was effectively removed while preserving biologically relevant signal. Further details and validation of this approach on the data used here can be found in [27].

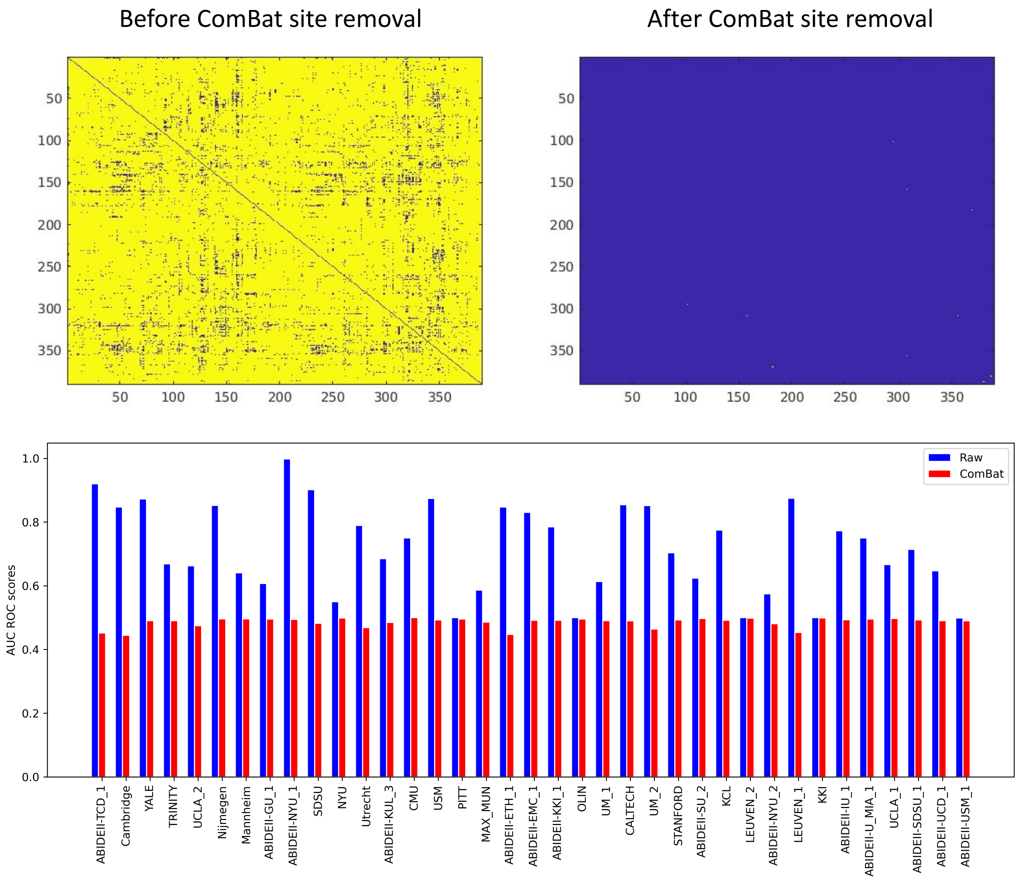

Figure S4. Evaluation of scanning site effects before and after ComBat harmonization.

Top panels show functional connectivity edges with significant (yellow, $p < 0.05$) and non-significant (blue, $p > 0.05$) site effects, tested for each pairwise connection. Before ComBat (left), the majority of connections show significant site effects; after ComBat (right), virtually none remain. Bottom panel shows area under the receiver operating characteristic curve (AUC ROC) scores from linear SVM one-vs-one classification of scanning sites. Before harmonization (blue), sites are readily distinguishable from the connectivity data (AUC ROC well above 0.5). After ComBat (red), classification performance drops to near chance across all site pairs, confirming effective removal of site-related variance.

Section 7. Normative modeling evaluation

We report distributions across connections of four metrics to evaluate the performance of the normative models (Figure S5). Explained variance quantifies the Pearson correlation between the predicted and the actual value at each edge. Most values fall within the range of r=0.1 and r=0.2, which is comparable to past normative modelling studies of fMRI data [28]. Mean standardized log loss (MSLL) measures the difference of the log loss of the test data and the log loss under the predicted distribution, standardized over the test data. Lower MSLL values indicate better model performance. The values in our model are close to 0 which indicates that the model performed well. Finally, skewness and kurtosis of the pseudo z-scores distributions measure how well the model distribution matches the observed distribution [29].The pseudo z-scores are quantiles of the observed distribution mapped to z-scores of a standard normal distribution [30]. The ideal skewness is 0 whereas the ideal kurtosis for normal distribution is 3, indicating that our model performs well also according to these metrics (Figure S5).

We additionally tested for age-by-sex interactions on pre-normative modelling FC estimates by fitting a linear model at each connection with age, sex and mean framewise displacement across all 75,855 connections. The analysis revealed only 2 significant interactions after FDR correction ($q < 0.05$), lending support for our use of an additive age, sex and mean framewise displacement model.

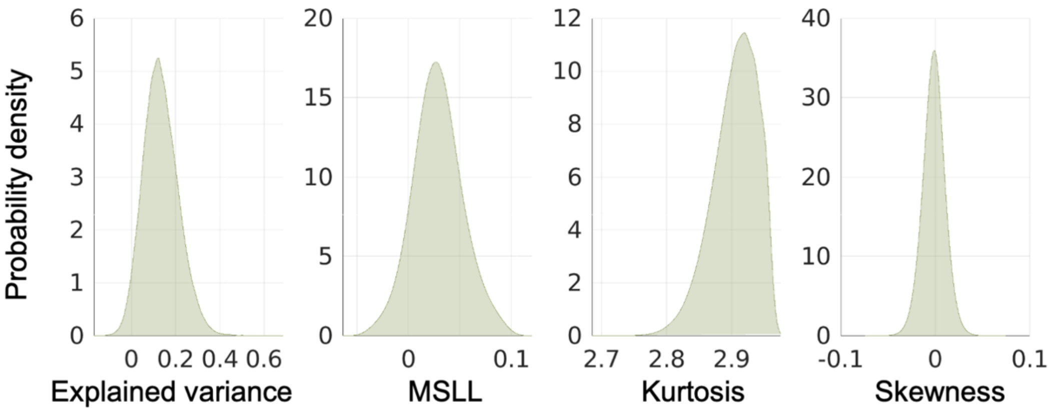

Figure S5. Distributions of the normative modelling evaluation metrics for the group of neurotypical individuals.

Explained variance by the normative model across edges (i.e., the proportion of variance in the target variable that is predictable from the input features, with higher values indicating better model fit); Mean Standardized Log Loss (MSLL) which compares the log loss of the model to that of a simple baseline predictor that always predicts the mean of the training data; Kurtosis and skew of the distribution of pseudo-z scores (values near 3 and near zero, respectively, indicate well-calibrated model estimates consistent with a standard normal distribution).

Section 8. Behavioral variables

Our analysis includes a range of behavioural measures essential for assessing various aspects of autism spectrum disorders (ASD). These include the Autism Diagnostic Observation Schedule (ADOS) calibrated severity scores

for social affect, communication, and restrictive and repetitive behaviours, and the Autism Diagnostic Interview-Revised (ADI-R) scores across the social, communication, and restricted and repetitive behaviours domains. Additionally, we utilized the scores from the Social Responsiveness Scale second edition (SRS-2) [31], The Short Sensory Profile (SSP) subscales [32] were also included to evaluate sensory processing difficulties, the Autism-Spectrum Quotient (AQ) [33] and full scale IQ assessed using the Wechsler Abbreviated Scales of Intelligence, Second Edition [34].

The ADI-R provides insights into an individual's developmental history through a structured interview conducted with parents or caregivers [35]. In contrast, the ADOS assesses current symptoms through a semi-structured interaction between the individual and an examiner [36,37]. For the purposes of this study, ADOS 2 scores from the ABIDE dataset were converted to calibrated severity scores [38,39] to maintain consistency with LEAP score methodologies.

The SRS-2 measures the presence and severity of social difficulties in autism, with assessments based on parent and/or teacher ratings for individuals under 18, and self-reports for those aged 18 and older [31].

The Short Sensory Profile (SSP) is a brief version of Dunn's Sensory Profile, a caregiver questionnaire designed to screen and identify children with sensory processing difficulties [32,40]. It comprises 38 items across seven subscales: Movement Sensitivity, Tactile Sensitivity, Sensation Seeking/Under-responsiveness, Visual/Auditory Sensitivity, Low Energy/Weak, Taste/Smell Sensitivity, and Auditory Filtering. To enhance the interpretability of the results, we inverted the SSP subscale scores such that lower scores now indicate less severe symptoms. To summarize the SSP in two variables, in our analysis we used summary measures of sensory hypo- and hypersensitivity calculated according to [41].

AQ [33] is a continuous measure, administered via self-report or parent-report, designed to quantify the extent of behavioural traits associated with Autism Spectrum Disorder (ASD) in children, adolescents, and adults of average intelligence.

Section 9. Results for different thresholds and parcellations

Our main analysis relied on a threshold of $|Z| > 2.3$ to define extreme thresholds, which is equivalent to $p = 0.01$. We used a fixed threshold because adaptive thresholds (e.g., the false discovery rate control), result in different thresholds being applied to different individuals and can complicate comparisons. To examine the sensitivity of our findings to this choice of threshold, we repeated our analyses applying the more liberal threshold $|Z|=1.7$ ($p = 0.05$) and the more conservative threshold of $|Z|=3.1$ ($p = 0.001$) to examine the overlap of participants across the 3 levels.

In line with our original analysis, no significant overlap difference was found on the level of individual connections for the two alternative thresholds. At the level of regions and networks, the results for $|Z|=1.7$ showed that subjects overlapped more in general (Figure S6 A) compared to our original analysis (Figure 3B), which is expected given the lower threshold. Statistical comparison of the overlaps between autistic and neurotypical individuals revealed a lower but more wide-spread relative difference for both positive and negative deviations, reaching up to 1,35 in some regions and 2,1 times more overlap in some networks compared to controls, exhibiting a similar pattern to our reported results on both region and network level.

The results for the much more stringent threshold of $|Z|=3.1$ revealed lower overlap across thresholds at connection, region, and network levels. We further found higher, but less wide-spread overlap differences for positive deviations at region and network levels, as well as for negative deviations at the region level. There were no significant group differences at the network level for negative deviations (Figure S6 B).

Additionally, we repeated the overlap analysis using a threshold of $|Z| > 2.6$, which has been employed in previous normative modeling studies of autism [42-44].The spatial pattern of group differences at both region and network levels was very similar to that obtained with our primary threshold of $|Z| > 2.3$, confirming that our findings are not driven by the specific threshold choice (Figure S7).

To assess whether our findings are robust to the choice of parcellation resolution, we repeated the main overlap analysis using both a coarser (Schaefer 200-region) and a finer (Schaefer 800-region) cortical parcellation. The spatial distribution of group differences in both positive and negative deviations was consistent across resolutions, reproducing the patterns observed in the primary Schaefer 400 analysis (Figure S8). This confirms that our results are not dependent on the granularity of the parcellation scheme.

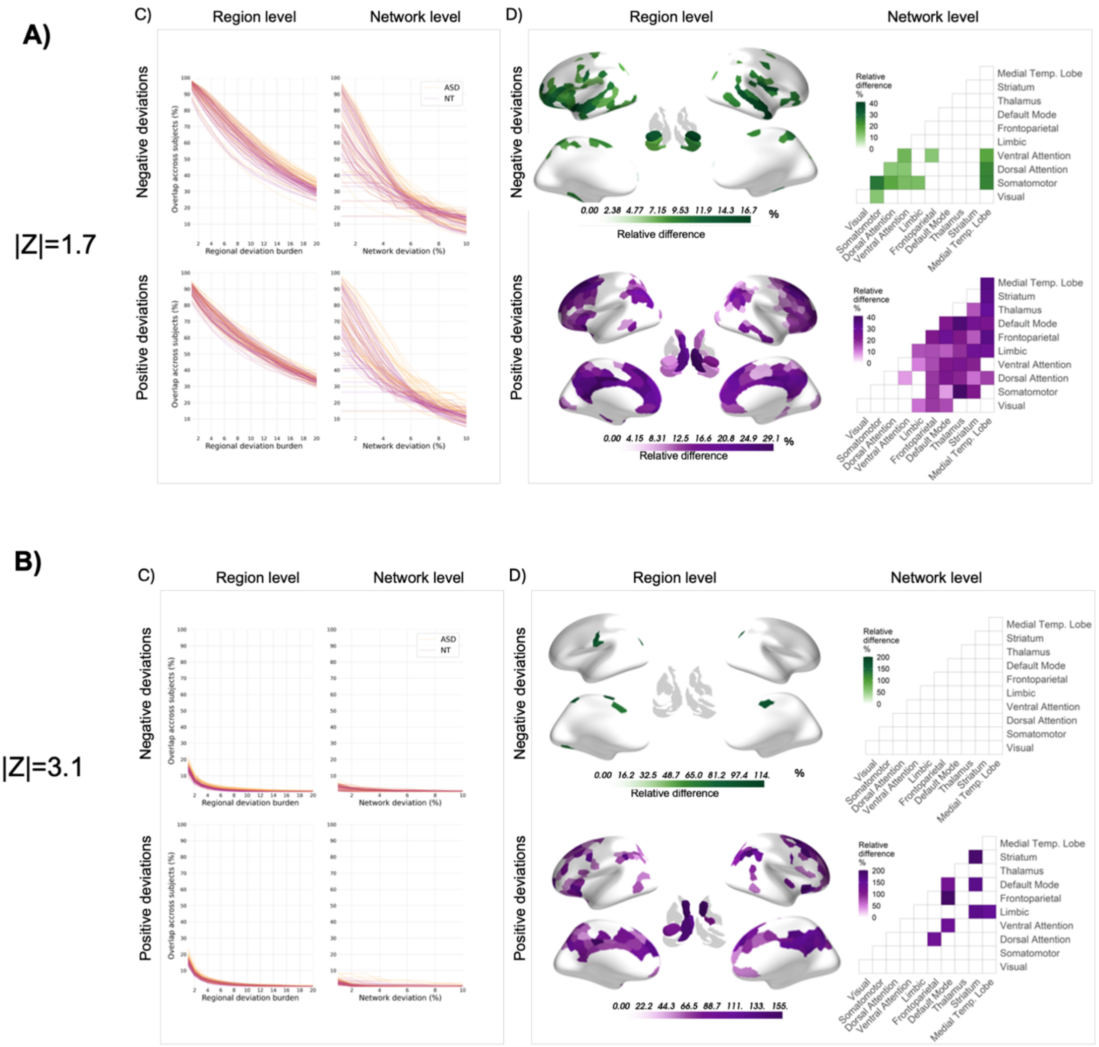

Figure S6. Overlap of negative and positive functional connectivity (FC) deviations at the level of regions, and networks for alternative thresholds.
A) Overlap across thresholds and group differences in overlap for extreme deviations defined at thresholds of |Z|=1.7 (p=0.05) and B) |Z|=3.1 (p=0.001), respectively. Line plots show the overlap of subjects at the level of regions and networks across different region and network deviation degree thresholds. Each line represents a region in the brain. Yellow color indicates overlap across autistic individuals, whereas red indicates overlap across neurotypical controls. The second column shows the overlap of negative and positive network deviation percent. Here, each line represents a within or between network overlap across participants, where yellow indicates overlap across the group of autistic individuals and red indicates the group of controls. D) Regions and networks showing significantly greater overlap in autistic individuals represented as relative differences. Green color indicates overlap of negative deviations; purple color indicates overlap of positive deviations.

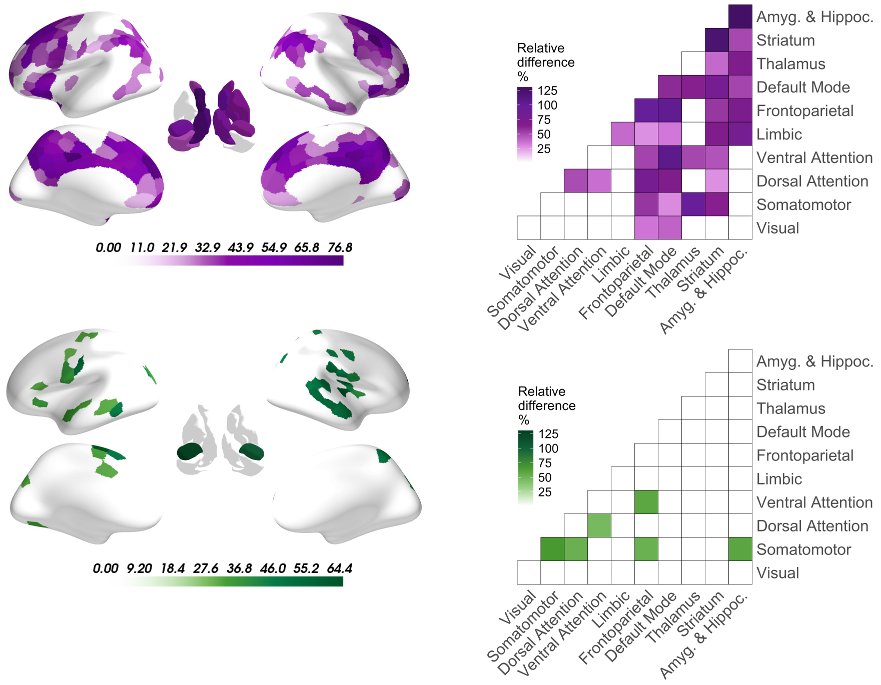

Figure S7. Threshold sensitivity analysis at |Z| > 2.6.
Overlap of extreme deviations between autistic and neurotypical groups using the threshold of |Z| > 2.6, as employed in previous normative modelling studies.

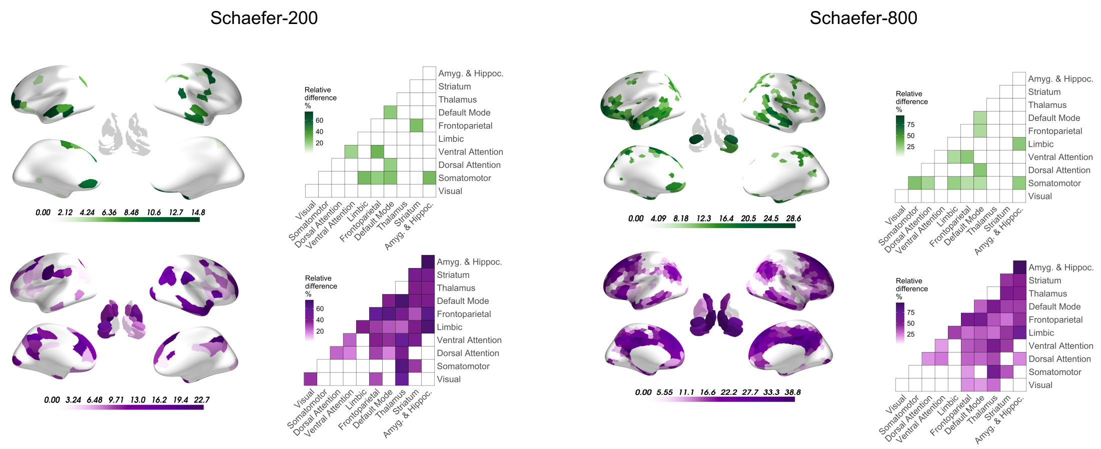

Figure S8. Parcellation resolution sensitivity analysis.
Overlap of extreme deviations between autistic and neurotypical groups repeated using the Schaefer 200-region (left) and Schaefer 800-region (right) cortical parcellations. Top rows (green) show negative deviations; bottom rows (purple) show positive deviations. For each parcellation, cortical surface maps display the relative significant difference of regional, and matrices show the relative difference in overlap between groups at the network level.

Section 10. Sensitivity analyses

In order to test the sensitivity of our results to participant's sex, attention deficit hyperactivity disorder (ADHD) comorbidity and psychotropic medication, we performed our analyses 1) including only male participants (655 autistic with mean age 16.4 years; 772 neurotypical with mean age 16.3 years; 2) excluding participants with ADHD comorbidity (651 autistic with average age of 16.9 years; 105 female; 1018 neurotypical with average age 15.9 years; 250 female) and 3) excluding all subjects that take psychotropic medication (591 autistic with average age of 16.7 years; 99 female; 1007 neurotypical with average age 15.9 years; 250 female). We report the Pearson correlation of the between group difference result from the sensitivity analyses with the original analysis between-group difference in Table S4, both on the network and region level.

Table S4. Pearson correlations of sensitivity analyses results with the original analysis result

	Networks		Regions	
	Negative deviations	Positive deviations	Negative deviations	Positive deviations
Only males	0.92	0.95	0.8	0.9
No ADHD comorbidity	0.99	0.98	0.8	0.93
Unmedicated	0.98	0.98	0.81	0.94

Section 11. Brain- behavior relationship

We used support vector regression (SVR) to develop multivariate predictive models of clinical and cognitive measures using FC deviation scores at the level of connections, regions, and networks. For each variable and FC resolution, we used five-fold cross-validation with 70% discovery 30% validation splits. We applied a principal component analysis (PCA) to the discovery data, retaining components that explained 50% of the variance, and trained a linear SVR, epsilon insensitive model using unthresholded deviation values to assess the connection-level relationships. Network and regional values were calculated on data with threshold applied at τ=2.3, as in the overlap analysis. Five-fold grid-search cross-validation was applied to optimize ε and C parameters according to the coefficient of determination. The held-out validation data were projected into the discovery data principal component space, to ensure that the model trained on the discovery set generalizes to unseen data. We next applied the linear SVR model on the PCA-projected validation data sets to predict the phenotypic scores in unseen data. The strength of the association in out-of-sample data was quantified by the correlation between the model's predicted and the observed phenotypic scores. We report the median correlation across the five folds and their interquartile range. Statistical significance was determined through 1,000 permutations per variable and FDR correction.

Section 12. Dataset Sensitivity and stability Analysis

To evaluate the robustness of our findings across different datasets, we conducted a sensitivity analysis by performing our primary analyses separately on two dataset subgroups: the LEAP data and the combined ABIDE 1 and 2 data. We then assessed the consistency of results by calculating Pearson correlations between the group difference maps obtained from each dataset subset and those from our main pooled analysis. These correlation coefficients, which quantify the spatial similarity of group differences across datasets, are presented in Supplementary Table S5. This approach allows us to determine whether our reported effects are driven by characteristics specific to individual datasets or represent consistent patterns across the broader sample.

To further assess the stability of our findings, we performed a bootstrap reliability analysis with 1,000 iterations, resampling participants with replacement within each group. For each iteration, we recomputed group difference maps and evaluated their consistency with the main results. At the network level, inter-bootstrap correlations indicated good reliability for positive deviations (mean r = 0.814) and acceptable reliability for negative deviations (mean r = 0.733). Region-level analyses showed a similar pattern (positive deviations mean r = 0.829; negative deviations mean r = 0.663). Analysis of the consistency of the direction of the group differences

further confirmed that most network pairs and regions maintained the same effect polarity across bootstrap samples, with mean consistency exceeding 88% for both positive and negative deviations at the region level, and with 100% consistency in significant networks (Figure S9). Together, these results indicate that both the magnitude and direction of our reported group differences are robust to sampling variability.

Table S5. Sensitivity to dataset

	Networks		Regions	
	Negative deviations	Positive deviations	Negative deviations	Positive deviations
LEAP	0.9	0.72	0.7	0.82
ABIDE 1 and 2	0.94	0.95	0.89	0.96

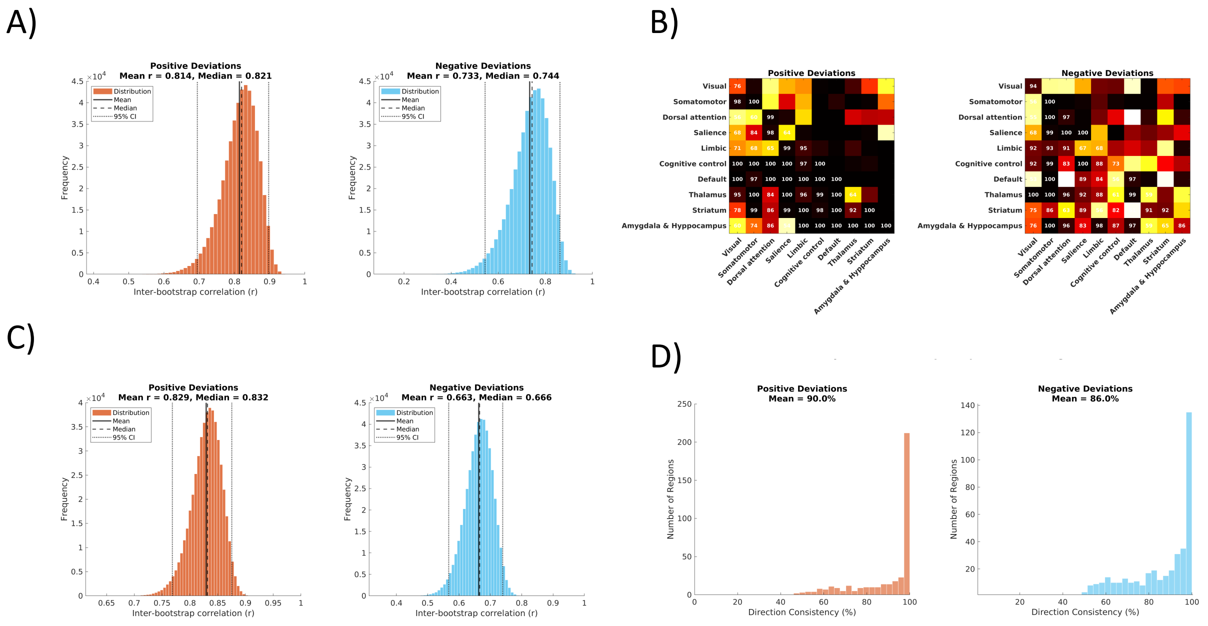

Figure S9. Bootstrap reliability analysis of network- and region-level deviation overlap.

A) Distribution of inter-bootstrap correlations for network-level analyses from 1,000 bootstrap iterations. Dashed lines indicate mean and median; dotted lines indicate 95% confidence intervals. B) Direction consistency matrices showing the percentage of bootstrap samples in which each network pair exhibited the same direction of effect (ASD > TD or TD > ASD) as the observed data. Values represent consistency percentages across 1,000 bootstrap iterations. Most network pairs showed high consistency (>80%), indicating stable effect directions. C) Distribution of inter-bootstrap correlations for region-level analyses (390 regions

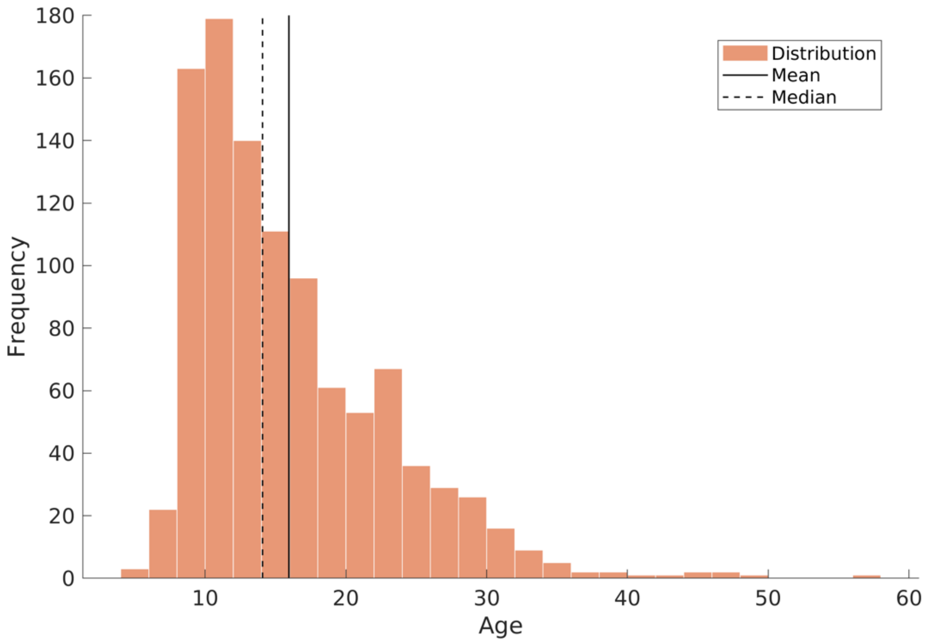

Figure S10. Age distribution of the group of neurotypical individuals

The findings are most robust within the 8–35 year range, where we have the most coverage in our training data. This range covers 93% of autistic individuals in our sample.

References

1. Loth E, Charman T, Mason L, et al. The EU-AIMS Longitudinal European Autism Project (LEAP): design and methodologies to identify and validate stratification biomarkers for autism spectrum disorders. *Mol Autism*. 2017;8:24. doi:10.1186/s13229-017-0146-8
2. Charman T, Loth E, Tillmann J, et al. The EU-AIMS Longitudinal European Autism Project (LEAP): clinical characterisation. *Mol Autism*. 2017;8:27. doi:10.1186/s13229-017-0145-9
3. Di Martino A, Yan CG, Li Q, et al. The autism brain imaging data exchange: towards a large-scale evaluation of the intrinsic brain architecture in autism. *Mol Psychiatry*. Jun 2014;19(6):659-67. doi:10.1038/mp.2013.78
4. Di Martino A, O'connor D, Chen B, et al. Enhancing studies of the connectome in autism using the autism brain imaging data exchange II. *Scientific data*. 2017;4(1):1-15.
5. Parkes L, Fulcher B, Yucel M, Fornito A. An evaluation of the efficacy, reliability, and sensitivity of motion correction strategies for resting-state functional MRI. *Neuroimage*. May 1 2018;171:415-436. doi:10.1016/j.neuroimage.2017.12.073
6. Satterthwaite TD, Elliott MA, Gerraty RT, et al. An improved framework for confound regression and filtering for control of motion artifact in the preprocessing of resting-state functional connectivity data. *Neuroimage*. Jan 1 2013;64:240-56. doi:10.1016/j.neuroimage.2012.08.052
7. Power. A simple but useful way to assess fMRI scan qualities. *Neuroimage*. 2017;154:150-158.
8. Aquino KM, Fulcher BD, Parkes L, Sabaroedin K, Fornito A. Identifying and removing widespread signal deflections from fMRI data: Rethinking the global signal regression problem. *NeuroImage*. 2020;212:116614.
9. Kundu P, Inati SJ, Evans JW, Luh W-M, Bandettini PA. Differentiating BOLD and non-BOLD signals in fMRI time series using multi-echo EPI. *Neuroimage*. 2012;60(3):1759-1770.
10. Smith SM, Jenkinson M, Woolrich MW, et al. Advances in functional and structural MR image analysis and implementation as FSL. *Neuroimage*. 2004;23 Suppl 1:S208-19. doi:10.1016/j.neuroimage.2004.07.051
11. Pruim RHR, Mennes M, van Rooij D, Llera A, Buitelaar JK, Beckmann CF. ICA-AROMA: A robust ICA-based strategy for removing motion artifacts from fMRI data. *Neuroimage*. May 15 2015;112:267-277. doi:10.1016/j.neuroimage.2015.02.064
12. Pruim RHR, Mennes M, Buitelaar JK, Beckmann CF. Evaluation of ICA-AROMA and alternative strategies for motion artifact removal in resting state fMRI. *Neuroimage*. May 15 2015;112:278-287. doi:10.1016/j.neuroimage.2015.02.063
13. Jenkinson M, Bannister P, Brady M, Smith S. Improved optimization for the robust and accurate linear registration and motion correction of brain images. *Neuroimage*. Oct 2002;17(2):825-41. doi:10.1016/s1053-8119(02)91132-8
14. Jenkinson M, Smith S. A global optimisation method for robust affine registration of brain images. *Med Image Anal*. Jun 2001;5(2):143-56. doi:10.1016/s1361-8415(01)00036-6
15. Ciric R, Wolf DH, Power JD, et al. Benchmarking of participant-level confound regression strategies for the control of motion artifact in studies of functional connectivity. *Neuroimage*. 2017;154:174-187.
16. Power JD, Barnes KA, Snyder AZ, Schlaggar BL, Petersen SE. Spurious but systematic correlations in functional connectivity MRI networks arise from subject motion. *Neuroimage*. Feb 1 2012;59(3):2142-54. doi:10.1016/j.neuroimage.2011.10.018
17. Satterthwaite TD, Wolf DH, Loughead J, et al. Impact of in-scanner head motion on multiple measures of functional connectivity: relevance for studies of neurodevelopment in youth. *Neuroimage*. 2012;60(1):623-632.
18. Tyszka JM, Kennedy DP, Paul LK, Adolphs R. Largely typical patterns of resting-state functional connectivity in high-functioning adults with autism. *Cerebral cortex*. 2014;24(7):1894-1905.

19. Llera A, Pruim R, Wiegerinck W, Beckmann C. Gaussian/Inverse Gamma mixture models of ICA maps. 2015:
20. Llera A, Huertas I, Mir P, Beckmann CF. Quantitative intensity harmonization of dopamine transporter SPECT images using gamma mixture models. *Molecular imaging and biology*. 2019;21(2):339-347.
21. Llera A, Vidaurre D, Pruim R, Beckmann C. Variational mixture models with gamma or inverse-gamma components. *arXiv preprint arXiv:160707573*. 2016;
22. Chauvin RJ, Mennes M, Buitelaar JK, Beckmann CF. Assessing age-dependent multi-task functional co-activation changes using measures of task-potency. *Developmental cognitive neuroscience*. 2018;33:5-16.
23. Chauvin RJ, Mennes M, Llera A, Buitelaar JK, Beckmann CF. Disentangling common from specific processing across tasks using task potency. *Neuroimage*. 2019;184:632-645.
24. Chauvin RJ, Buitelaar JK, Sprooten E, et al. Task-generic and task-specific connectivity modulations in the ADHD brain: an integrated analysis across multiple tasks. *Translational psychiatry*. 2021;11(1):1-10.
25. Johnson WE, Li C, Rabinovic A. Adjusting batch effects in microarray expression data using empirical Bayes methods. *Biostatistics*. 2007;8(1):118-127.
26. Yu M, Linn KA, Cook PA, et al. Statistical harmonization corrects site effects in functional connectivity measurements from multi-site fMRI data. *Human brain mapping*. 2018;39(11):4213-4227.
27. Ilioska I, Oldehinkel M, Llera A, et al. Connectome-wide mega-analysis reveals robust patterns of atypical functional connectivity in autism. *medRxiv*. 2022;
28. Rutherford S, Barkema P, Tso IF, et al. Evidence for embracing normative modeling. *Elife*. 2023;12:e85082.
29. Dinga R, Fraza CJ, Bayer JM, Kia SM, Beckmann CF, Marquand AF. Normative modeling of neuroimaging data using generalized additive models of location scale and shape. *BioRxiv*. 2021:2021.06. 14.448106.
30. Dunn PK, Smyth GK. Randomized quantile residuals. *Journal of Computational and graphical statistics*. 1996;5(3):236-244.
31. Constantino JN, Gruber CP. *Social responsiveness scale: SRS-2*. Western Psychological Services Torrance, CA; 2012.
32. McIntosh D, Miller L, Shyu V, Dunn W. Development and validation of the short sensory profile. *Sensory profile manual*. 1999:59-73.
33. Baron-Cohen S, Wheelwright S, Skinner R, Martin J, Clubley E. The autism-spectrum quotient (AQ): Evidence from asperger syndrome/high-functioning autism, malesand females, scientists and mathematicians. *Journal of autism and developmental disorders*. 2001;31:5-17.
34. Wechsler D. Wechsler abbreviated scale of intelligence. 1999;
35. Rutter M, Le Couteur A, Lord C. Autism diagnostic interview-revised. *Los Angeles, CA: Western Psychological Services*. 2003;29(2003):30.
36. Lord C, Rutter M, DiLavore PC, Risi S, Gotham K, Bishop SL. ADOS. *Autism diagnostic observation schedule Manual Los Angeles: WPS*. 1999;
37. Reaven JA, Hepburn SL, Ross RG. Use of the ADOS and ADI-R in children with psychosis: importance of clinical judgment. *Clinical child psychology and psychiatry*. 2008;13(1):81-94.
38. Hus V, Gotham K, Lord C. Standardizing ADOS domain scores: Separating severity of social affect and restricted and repetitive behaviors. *Journal of autism and developmental disorders*. 2014;44(10):2400-2412.
39. Hus V, Lord C. The autism diagnostic observation schedule, module 4: revised algorithm and standardized severity scores. *Journal of autism and developmental disorders*. 2014;44(8):1996-2012.
40. Dunn W. *Sensory profile: User's manual*. Psychological Corporation San Antonio, TX; 1999.

41. Lefebvre A, Tillmann J, Cliquet F, et al. Tackling hypo and hyper sensory processing heterogeneity in autism: From clinical stratification to genetic pathways. *Autism Res*. Feb 2023;16(2):364-378. doi:10.1002/aur.2861
42. Wolfers T, Beckmann CF, Hoogman M, Buitelaar JK, Franke B, Marquand AF. Individual differences v. the average patient: mapping the heterogeneity in ADHD using normative models. *Psychological Medicine*. 2020;50(2):314-323.
43. Sun X, Sun J, Lu X, et al. Mapping Neurophysiological Subtypes of Major Depressive Disorder Using Normative Models of the Functional Connectome. *Biol Psychiatry*. Dec 15 2023;94(12):936-947. doi:10.1016/j.biopsych.2023.05.021
44. Liu Q, Lai H, Le J, et al. Identifying brain functional subtypes and corresponding task performance profiles in autism spectrum disorder. *Molecular Psychiatry*. 2025/11/01 2025;30(11):5034-5044. doi:10.1038/s41380-025-03086-x
