# Supplementary material for: Multiscale heterogeneity of atypical functional connectivity in autism
Source: Nat Ment Health. 2026 Jun 1;4(6):1010–20. doi: 10.1038/s44220-026-00656-y (PMC13259941; doi:10.1038/s44220-026-00656-y)
Supplement: Supplementary file 2 — Reporting summary [file 44220_2026_656_MOESM2_ESM.pdf]

nature portfolio

Corresponding author(s): Iva Ilioska

Last updated by author(s): Feb 23, 2026

Reporting Summary

Nature Portfolio wishes to improve the reproducibility of the work that we publish. This form provides structure for consistency and transparency in reporting. For further information on Nature Portfolio policies, see our Editorial Policies and the Editorial Policy Checklist.

Statistics

For all statistical analyses, confirm that the following items are present in the figure legend, table legend, main text, or Methods section.

n/a	Confirmed	
☐	☒	The exact sample size (*n*) for each experimental group/condition, given as a discrete number and unit of measurement
☐	☒	A statement on whether measurements were taken from distinct samples or whether the same sample was measured repeatedly
☐	☒	The statistical test(s) used AND whether they are one- or two-sided<br>*Only common tests should be described solely by name; describe more complex techniques in the Methods section.*
☐	☒	A description of all covariates tested
☐	☒	A description of any assumptions or corrections, such as tests of normality and adjustment for multiple comparisons
☐	☒	A full description of the statistical parameters including central tendency (e.g. means) or other basic estimates (e.g. regression coefficient) AND variation (e.g. standard deviation) or associated estimates of uncertainty (e.g. confidence intervals)
☐	☒	For null hypothesis testing, the test statistic (e.g. *F*, *t*, *r*) with confidence intervals, effect sizes, degrees of freedom and *P* value noted<br>*Give P values as exact values whenever suitable.*
☒	☐	For Bayesian analysis, information on the choice of priors and Markov chain Monte Carlo settings
☒	☐	For hierarchical and complex designs, identification of the appropriate level for tests and full reporting of outcomes
☐	☒	Estimates of effect sizes (e.g. Cohen's *d*, Pearson's *r*), indicating how they were calculated

Our web collection on statistics for biologists contains articles on many of the points above.

Software and code

Policy information about availability of computer code

Data collection	We used resting state fMRI and clinical variables collected by the EU-AIMS LEAP consortium and the ABIDE1 and 2 initiatives.
Data analysis	FMRIB Software Library 5.0.10, Matlab R2018b, Spyder(Python 3.12), Predictive Clinical Neuroscience Toolkit 0.20, scikit-learn 0.24.2 code for the analyses is available at https://github.com/ivaili/MultiscaleHeterogeneity

For manuscripts utilizing custom algorithms or software that are central to the research but not yet described in published literature, software must be made available to editors and reviewers. We strongly encourage code deposition in a community repository (e.g. GitHub). See the Nature Portfolio guidelines for submitting code & software for further information.

Data

Policy information about availability of data

All manuscripts must include a data availability statement. This statement should provide the following information, where applicable:

- Accession codes, unique identifiers, or web links for publicly available datasets
- A description of any restrictions on data availability
- For clinical datasets or third party data, please ensure that the statement adheres to our policy

Data collected within the ABIDE 1&2 initiatives is available for public use on the following links http://fcon_1000.projects.nitrc.org/indi/abide/abide_I.html; http://fcon_1000.projects.nitrc.org/indi/abide/abide_II.html .
Data from LEAP are stored at the EU-AIMS database at the Pasteur Institute in Paris. LEAP data is only accessible to consortium members with an analysis proposal

approved, and it will be available for use to the wider research public through open-access publication via a secure database that will become available in the near future (https://elixir-luxembourg.org/).

Research involving human participants, their data, or biological material

Policy information about studies with human participants or human data. See also policy information about sex, gender (identity/presentation), and sexual orientation and race, ethnicity and racism.

Reporting on sex and gender	For participants in the EU-AIMS LEAP consortium, sex was initially determined by self- or parent report and subsequently confirmed through DNA analysis. For participants in the ABIDE initiatives, sex was determined via self- or parent report.<br><br>Sex was included as a covariate in all normative modeling analyses. To assess whether our findings were driven by sex-specific effects, we performed a sensitivity analysis restricting our sample to male participants only. This male-only analysis yielded results that were highly consistent with our main findings, demonstrating that our reported effects are robust to participants' sex and not attributable to sex-related differences in brain structure or function.
Reporting on race, ethnicity, or other socially relevant groupings	In the EU-AIMS LEAP cohort, self-identified race was collected via parental- or self-report and categorized as White, Asian, Black, Mixed, or Other, following the classification system adopted by the participating clinical sites, numbers can be found in Table 1. For the ABIDE I and II datasets, race or ethnicity was not collected as part of the shared phenotypic protocol. ABIDE is a retrospective, multi-site databank aggregated from previously and independently collected datasets drawn from multiple international sites across North America and Europe. Demographic harmonization was limited to variables consistently available across all contributing sites (i.e., age, sex, handedness, and IQ) and race/ethnicity was not among them.
Population characteristics	796 autistic individuals (141 females; age-range: 5-58), 1028 neurotypical controls (256 females; age-range: 5-56)
Recruitment	For autistic participants: At each study site, participants with ASD and mild ID are recruited from existing local databases, clinic contacts, and local and national support groups.<br>For typically developing participants: participants are recruited via mainstream schools, flyers (e.g. left at youth centres, colleges, churches, etc.), and existing databases
Ethics oversight	The study was approved by national and local ethics review boards at each study site and is carried out to Good Clinical Practice (ICH GCP) standards.

Note that full information on the approval of the study protocol must also be provided in the manuscript.

Field-specific reporting

Please select the one below that is the best fit for your research. If you are not sure, read the appropriate sections before making your selection.

☒ Life sciences ☐ Behavioural & social sciences ☐ Ecological, evolutionary & environmental sciences

For a reference copy of the document with all sections, see nature.com/documents/nr-reporting-summary-flat.pdf

Life sciences study design

All studies must disclose on these points even when the disclosure is negative.

Sample size	All available participant data that passed our quality control criteria was included from the 3 datasets. No previous sample size calculations were performed.
Data exclusions	Of the 2,635 participants with available demographic and resting-state fMRI data across the three datasets (LEAP: 623; ABIDE I: 1,110; ABIDE II: 902), 811 (30.8%) were excluded through sequential quality control steps. Exclusions were made for preprocessing failure (N=25), incomplete scans (N=225), excessive head motion (N=426; mFD > 0.25mm, >20% of framewise displacements above 0.2mm, or any single displacement > 5mm), poor signal coverage (N=20), visible artifacts identified through carpet plot inspection (N=36), IQ below 70 (N=75), and structural brain abnormalities (N=4). The final sample comprised 1,824 participants (LEAP: 422; ABIDE I: 850; ABIDE II: 552), including 796 autistic individuals (141 female) and 1,028 neurotypical individuals (256 female), aged 5–58 years, recruited across 32 sites.
Replication	To assess the generalizability of our findings across different data sources, we conducted sensitivity analyses by repeating our primary analyses separately within the EU-AIMS LEAP dataset and the combined ABIDE 1 and 2 datasets. The results from these independent dataset analyses were highly consistent with our main findings from the pooled sample, confirming that our reported effects are not driven by dataset-specific characteristics and represent robust patterns that generalize across multiple independent cohorts.
Randomization	Participants were allocated into diagnostic groups depending on autism diagnosis
Blinding	We only used fMRI data and clinical variables. This data analysis and collection did not require blinding.

Reporting for specific materials, systems and methods

We require information from authors about some types of materials, experimental systems and methods used in many studies. Here, indicate whether each material, system or method listed is relevant to your study. If you are not sure if a list item applies to your research, read the appropriate section before selecting a response.

Materials & experimental systems

n/a	Involved in the study
☒	☐ Antibodies
☒	☐ Eukaryotic cell lines
☒	☐ Palaeontology and archaeology
☒	☐ Animals and other organisms
☒	☐ Clinical data
☒	☐ Dual use research of concern
☒	☐ Plants

Methods

n/a	Involved in the study
☒	☐ ChIP-seq
☒	☐ Flow cytometry
☐	☒ MRI-based neuroimaging

Plants

Seed stocks

Report on the source of all seed stocks or other plant material used. If applicable, state the seed stock centre and catalogue number. If plant specimens were collected from the field, describe the collection location, date and sampling procedures.

Novel plant genotypes

Describe the methods by which all novel plant genotypes were produced. This includes those generated by transgenic approaches, gene editing, chemical/radiation-based mutagenesis and hybridization. For transgenic lines, describe the transformation method, the number of independent lines analyzed and the generation upon which experiments were performed. For gene-edited lines, describe the editor used, the endogenous sequence targeted for editing, the targeting guide RNA sequence (if applicable) and how the editor was applied.

Authentication

Describe any authentication procedures for each seed stock used or novel genotype generated. Describe any experiments used to assess the effect of a mutation and, where applicable, how potential secondary effects (e.g. second site T-DNA insertions, mosiacism, off-target gene editing) were examined.

Magnetic resonance imaging

Experimental design

Design type: resting state fMRI

Design specifications: We used only resting state data.

Behavioral performance measures: No behavioral measures were collected during the recording

Acquisition

Imaging type(s): Functional imaging

Field strength: 3T

Sequence & imaging parameters: The scanning parameter details for each site is available in tables S1, S2 and S3 in the supplement.

Area of acquisition: Whole brain

Diffusion MRI: ☐ Used ☒ Not used

Preprocessing

Preprocessing software: FSL 5.0.10 was used for every step of the preprocessing.

Normalization: Participant functional images were co-registered to their respective anatomical images using boundary-based registration in FSL FLIRT. High-resolution structural images were registered to MNI152 standard space using a 12-parameter affine transformation and further refined with a non-linear registration via FSL FNIRT, employing a 10mm warp and 2mm resampling resolution. Finally, functional images were normalized to 2mm MNI152 standard space by applying the transformation of the functional image to T1 and T1 to MNI152. All subsequent analyses were conducted in MNI152 standard space.

Normalization template: MNI152 standard template

Noise and artifact removal: The following pipeline was implemented: removal of the initial five volumes for signal equilibration, volume realignment to the median volume using MCFLIRT for primary head motion correction, grand mean scaling, and spatial smoothing employing a 6mm FWHM Gaussian kernel. ICA-AROMA was then used for secondary head motion-related artifact correction. ICA-

	AROMA is capable of effectively removing motion-related artifacts while preserving neurobiological signals of interest, and has compares favorably to alternative methods for motion-related confound removal. Mean signals from the CSF and white matter were regressed out as nuisance covariates, and a 0.01Hz temporal high-pass filter was applied.
Volume censoring	No volume censoring was used. We excluded participants that did not meet the stringent motion-artefact-related quality control criteria

Statistical modeling & inference

Model type and settings	Statistical inference was done in a mass univariate manner, where we determined the difference of overlap between groups for each connection, region and network and we compared the difference to a distribution of 10, 0000 diagnostic label-shuffled permutations.
Effect(s) tested	We tested the difference in overlap of normative modeling deviations at the level of connections, regions and networks.

Specify type of analysis: ☒ Whole brain ☐ ROI-based ☐ Both

Statistic type for inference (See Eklund et al. 2016)	Statistical inference was done on the level of individual connections, regions and networks.
Correction	FDR statistical correction was applied to all p-values at each level of inference

Models & analysis

n/a	Involved in the study
☐	☒ Functional and/or effective connectivity
☒	☐ Graph analysis
☐	☒ Multivariate modeling or predictive analysis

Functional and/or effective connectivity	Pearson correlation was used to determine functional connectivity between regional pairs.
Multivariate modeling and predictive analysis	We used support vector regression to predict clinical variables from deviation maps at each level (connections, regions, networks)
