# Supplementary material for: Multiscale heterogeneity of atypical functional connectivity in autism
Source: Nat Ment Health. 2026 Jun 1;4(6):1010–20. doi: 10.1038/s44220-026-00656-y (PMC13259941; doi:10.1038/s44220-026-00656-y)
Supplement: Supplementary file 3 — Peer Review File [file 44220_2026_656_MOESM3_ESM.pdf]

nature portfolio

Peer Review File

Multiscale heterogeneity of atypical functional connectivity in autism

Corresponding Author: Dr Iva Ilioska

Parts of this Peer Review File have been redacted as indicated to maintain the confidentiality of unpublished data.

This file contains all reviewer reports in order by version, followed by all author rebuttals in order by version.

Version 0:

Reviewer comments:

Reviewer #1

(Remarks to the Author)
Ilioska et al. Explored heterogeneity of functional connectivity in an autistic sample by making use of a normative modeling approach. Connectivity was explored to the level of individual connections, regions, and networks. Dysfunctional connectivity was defined as a binary, as any value falling below a given value relative to the normative control sample would be marked as impaired.

This approach breaks assumptions of linear relationships between connections and phenotypes are complex behavior. Most importantly, by exploring the regional the network level, the authors are not assuming that the same set of connections will be impaired across all individuals with the given set of diagnosis, thus better accounting for individual variability and heterogeneity in clinical samples.

This approach has been used in prior work by the authors using different modalities, but represents a significant and novel break from standard analysis approaches.

Generally speaking, the approach is excellent, the work is topical, and it fits well with ongoing work from this research group. This approach provides opportunities to overcome challenges that have been noted in brainwide association studies, which assume common sets of impaired networks and linear relationships between FC or brain activity and complex behaviors or phenotypes. I also appreciate that results for higher and lower Z-threshold are presented, considerations of motion, and the sensitivity analyses.

I would consider this paper overall to be excellent. My primary concern relates to how the methods are presented. I appreciate the challenge of condensing such a study to the word count required by the journal, but generally speaking I found the methods extremely opaque. I was unable to understand specifically what was done in many cases, essentially having to make guesses or assumptions based on what I know from the author's prior work. I will lay out some specific cases where I feel the methods need to be clarified, but generally speaking, substantially more detail and clarification needs to be added to the methods, much of which could be potentially put in supplemental material.

The samples are well described, largely in the supplement, as is the imaging processing.

The gaussian gamma mixture model approach could be briefly expanded upon, as many readers will not be familiar with this approach, this reviewer included.

The tenfold cross validation used validate the model in the normative model section and supplemental section 7 is not well explained. I guess the r values in section 7 are the predicted versus actual on the left out fold? Please clarify. Relatedly Supp figure 3, graphs are not labeled. I can only assume the graphs are describing what is in the captions separated by semi-colons; this is not the ideal way to present data.

Notation $OR, G(\tau)$ is not well defined, and doesn't really add anything to the paper as it’s never referred to again. Honestly, in this regional analysis section I really don't understand at all what's being done, or how or why there are thresholds. What is

the threshold of zero mean? I can only guess what's actually being measured here, but I would have assumed that since the connections are defined as 0 or 1 for deviant or not, that it would simply be a count of how many connections from that region fell into the deviant range. Likewise I'm not sure what the area under the curve is measuring. This section is very unclear, I would have assumed it was total number of deviant connections per node but it looks like perhaps the number of people that have an exact match of which connections are deviant from which regions, Or a certain number of overlap? Very confused.

Same for the following section on network analysis, with 1 to 10%. It's not clear to me why overlap across participants would be more sensible than simply counting the connections, or how that overlap is actually calculated. Please clarify.

Figure 2 helps explain possibly some aspects of the methods, but it's still not clear why a threshold would be applied to count the number of participants, why this is called overlap, and what the AUC measures. Some of the confusion may stem from unclear use of the term 'overlap'.

The results are highly compelling and generally well presented, saving that they're hard to contextualize because the methods are unclear.

Figure 3 D caption And graph labels, ‘relative differences’ should be clarified, e.g. Is it the percent AD minus Percent NT?

The behavioral predictions analysis was quite interesting, and highlights how different levels of analysis may map on the different behavioral findings. I enjoyed the discussion of this as well.

The discussion is generally very good, though it relies a lot on a small number of prior papers from this group. It might be better contextualized in the broader literature, for example these results fit quite well with the lesion network model championed by these results fit quite well with the lesion network modelling approach championed by Fox and Siddiqi, showing that disparate regional findings can map onto a common Network.

The only potential additional analysis I might like to see is some sort of estimate of stability or overlap of findings if the data was sub-sampled. The sensitivity analysis across data sets helped a lot in this regard, but it would be interesting to see if various splits of the data showed similar patterns in the regional and Network levels. This is of potential import given the heterogeneity seen across studies, which this paper is attempting to address.

Overall an excellent paper. The conclusions are appropriate to the data, the use of statistics appears robust, and the work is timely given awareness of issues and heterogeneity and challenges that the brainwide associations observed at the connection level.

(Remarks on code availability)
I did not see any reference to provided code?

Reviewer #2

(Remarks to the Author)
This manuscript investigates the critical challenge of inter-individual heterogeneity in autism by examining atypical functional connectivity (FC) across multiple scales of brain organization. Utilizing a large, multi-site resting-state fMRI dataset (N=1824) from the EU-AIMS LEAP and ABIDE consortia, the authors employ normative modeling to quantify individual deviations from expected FC patterns, controlling for age, sex, and head motion.
The study reveals that FC heterogeneity in autism is scale-dependent. At the level of individual connections, deviations were highly idiosyncratic, with minimal overlap across participants (<4%), and the overall burden of deviations was similar between the autistic and neurotypical (NT) groups. However, at coarser scales (regions and networks), deviations in the autistic group showed significantly higher convergence. Specifically, the results highlight convergent hypoconnectivity (negative deviations) within sensorimotor and attention systems, and hyperconnectivity (positive deviations) involving the frontoparietal and default mode networks. These deviation patterns showed modest but statistically significant associations with clinical and cognitive abilities.
This is an excellent study characterized by exceptionally neat technical practices and sophisticated analytical methods. The authors are to be congratulated on this impressive work. The utilization of a large aggregated dataset, rigorous preprocessing, careful harmonization across 32 sites, and the implementation of Gaussian Process Regression (GPR) for normative modeling are highly laudable. The multiscale approach offers a compelling neurobiological framework for reconciling shared diagnostic criteria with extensive phenotypic variability, moving the field beyond the limitations of traditional case-control comparisons.
I support its publication pending revisions addressing the comments detailed below, which aim to enhance the clarity, robustness, and interpretation of these important findings.

Major Comments
1. The concept of "multiscale" analysis is central to the manuscript. While the approach is visualized (Figures 1 and 2), the theoretical motivation and rationale for specific methods could be articulated more clearly, particularly for a broad audience that includes clinicians. Please expand the Introduction to better elaborate on the rationale for expecting differences in heterogeneity across scales (connection, region, network). Why might heterogeneity decrease as the spatial scale coarsens? Articulating the theoretical underpinnings (e.g., diverse etiological pathways converging on common functional systems) will help readers interpret the significance of the observed scale-dependent heterogeneity.

2. Further, please provide brief justifications in the main text Methods section for key analytical decisions. For instance (not limited to), provide a brief rationale for the use of Gaussian-gamma mixture modeling (e.g., its advantage in enhancing differentiation of signal from noise). Also, please clarify the rationale for the specific choice of $|Z|>2.3$ ($p=0.01$) as the primary threshold for extreme deviations.

3. The analyses are based on the Schaefer 400-region cortical parcellation (plus subcortical ROIs). As the definition of nodes in connectomics inherently influences the resulting metrics, it is crucial to demonstrate that the findings are robust to the specific resolution of the chosen parcellation. The authors should repeat the main overlap analysis (reported in Figure 3) using different parcellation resolutions (e.g., a coarser Schaefer 200 and a finer Schaefer 800 atlas).

4. The authors include sex as a covariate in the GPR model. This approach typically models sex as an additive effect, assuming that the trajectory of FC across age is parallel between males and females. Given the established sex differences in neurodevelopmental trajectories and autism, this assumption may not hold. Modeling sex merely as a covariate, without accounting for interactions (e.g., age-by-sex), can lead to inaccurate norms and potentially bias the resulting deviation scores. It is recommended to formally test for age-by-sex interactions. If significant interactions are present, or if model fit differs substantially between sexes, the normative models should ideally be stratified (trained separately for males and females) to ensure accurate modeling, although the constraints of the female sample size are acknowledged.

5. The study covers a very wide age range (5-58 years). Normative models are typically least stable and most uncertain at the extremes of the covariate distribution where data may be sparse. To assess the reliability of the model across the lifespan, please provide a visualization (e.g., histogram or density plot) of the age distribution for the training sample (NT controls). Please visualize or discuss the uncertainty of the predictions (e.g., the predictive variance from the GPR) across the age range, particularly at the younger and older ends.

6. As detailed in the Supplement (Section 2), participants with IQ < 70 were excluded. Table 1 confirms the resulting sample is high-functioning (Mean IQ Autism ≈ 106). This significantly limits the generalizability of the findings to the entire autism spectrum, which includes individuals with intellectual disabilities. This limitation must be explicitly addressed in the main text's Limitations section, and the conclusions should reflect the specific population studied.

7. The multivariate predictive models (Figure 4) show statistically significant correlations, but the effect sizes are modest (median correlations ranging from 0.1 to 0.29). The discussion and abstract (e.g., "representing viable targets for biomarker... development") should be tempered to reflect the modest strength of these associations and the challenges remaining before clinical translation.

8. The authors conclude that connection-level heterogeneity is within "normative expectations" because the overlap in the autistic group is similar to the control group. However, the estimation procedures differ: control deviations were quantified using 10-fold cross-validation (within the training data), whereas autism deviations were quantified by applying the NT-trained model to the held-out autism data. The authors should discuss the nuances of comparing deviations derived from a test set (autism) versus a cross-validated training set (controls) when interpreting the baseline level of heterogeneity.

9. Table 1 indicates a significant difference in mean FD between the groups ($p < .0001$). While the authors employed rigorous denoising (validated in Supplement Section 4) and included mean FD in the normative model, two concerns remain. First, to confirm that motion effects were successfully modeled as a covariate, please report the correlation between the final deviation scores (Z-scores) or the total number of extreme deviations per participant and mean FD for both groups. Second, given that the findings highlight significant convergence in the sensorimotor network—an area susceptible to motion artifacts—the authors should briefly discuss the spatial topography of their findings and argue why these results are likely neurobiological rather than residual artifacts.

Minor Comments
1. For a multi-site study of this scale, please provide diagnostics showing the efficacy of ComBat harmonization (e.g., variance explained by site before and after harmonization) in the Supplement.

2. Reference 10 (Segal A, Parkes L, Aquino K, et al. medRxiv. 2022) has been published. Please update the citation.

3. Please check for citation consistency throughout the text. There are frequent instances of spaces between the text and the citation.

4. Line 176: "...with the upper bound of 10% chose because..." should be "chosen".

5. There is a discrepancy in the final sample sizes reported. The main text reports 796 autistic and 1028 NT individuals. The Supplement (Section 3, p. 4, last paragraph) reports 773 autistic individuals and 994 NT individuals. Please clarify this discrepancy.

6. Please review the supplements for minor typos.

(Remarks on code availability)

Reviewer #3

(Remarks to the Author)
While the present study applied normative modeling to quantify heterogeneity in functional connectivity in ASD, the methodological approach was not novel(DOI: 10.1038/s41380-025-03086-x; DOI: 10.1016/j.biopsych.2023.05.021.). The current findings just established patterns of heterogeneity without providing substantial new evidence or a novel theoretical perspective.

Introduction
(1) Lines 91-93: Normative modeling has revealed not only structure but function deviations. Please add more details. For example, DOI: 10.1038/s41380-025-03086-x; DOI: 10.1016/j.biopsych.2023.05.021.
(2) Line 95: Please specific multiscale normative modeling in the introduction section.
(3) Line 153,163: In contrast to previous studies that used a stringent threshold (e.g., Z = ±2.58, 2.6) for deviation maps (Sun et al., 2023; Liu et al., 2025; Wolfers et al., 2018; Wolfers et al., 2020), the current study employed a more liberal threshold of Z = ±2.3. This less conservative approach captures a broader range of deviations.
Ref:https://doi.org/10.1001/ jamapsychiatry.2018.2467;https://doi.org/ 10.1017/S0033291719000084.

Results
(4) Line 207: I am confused about this statement “These findings indicate that autism is associated with expected levels of extreme FC deviations when examined at the level of specific pairwise connections.” How can authors get this finding from the results?
(5) Line 246-254: What about the correlations between the true and predicted values if combined the features of three levels.

(Remarks on code availability)
The authors did not provide any code.

Version 1:

Reviewer comments:

Reviewer #1

(Remarks to the Author)
I would like to congratulate the authors on an excellent paper and well done response. I have no further major comments.

I found a minor typo:
Page 8 , line 228
“region level, , deviation degree”

Also the code git repo needs a readme explaining how to run the analysis. And while some code was shared it represents only a fraction of the analysis, has minimal comments, and loads data in an unknown format and thus is hard to read and replicate. Though some of the process is clear (ish) as the code is not especially dense (simple is good).

I look forward to seeing future work in this direction.

(Remarks on code availability)
Repeated from author comments:

Also the code git repo needs a readme explaining how to run the analysis. And while some code was shared it represents only a fraction of the analysis, has minimal comments, and loads data in an unknown format and thus is hard to read and replicate. Though some of the process is clear (ish) as the code is not especially dense (simple is good).

Its very bare bones and only some of the calculations for outcomes.

Reviewer #2

(Remarks to the Author)
I appreciate that the authors have addressed all of my comments carefully. I am happy to endorse the publication of this work in its current form. Congratulations to this beautiful work!

(Remarks on code availability)
The code is neat and sufficient to generate the reproducible pipelines.

Reviewer #3

(Remarks to the Author)
The authors have addressed all my concerns.

(Remarks on code availability)
The current code is sufficient for reproducing these results in the manuscript.

Open Access This Peer Review File is licensed under a Creative Commons Attribution 4.0 International License, which permits use, sharing, adaptation, distribution and reproduction in any medium or format, as long as you give appropriate credit to the original author(s) and the source, provide a link to the Creative Commons license, and indicate if changes were made.
In cases where reviewers are anonymous, credit should be given to 'Anonymous Referee' and the source.
The images or other third party material in this Peer Review File are included in the article's Creative Commons license, unless indicated otherwise in a credit line to the material. If material is not included in the article's Creative Commons license and your intended use is not permitted by statutory regulation or exceeds the permitted use, you will need to obtain permission directly from the copyright holder.
To view a copy of this license, visit https://creativecommons.org/licenses/by/4.0/

Reviewer #1:

Comment 1: Ilioska et al. Explored heterogeneity of functional connectivity in an autistic sample by making use of a normative modeling approach. Connectivity was explored to the level of individual connections, regions, and networks. Dysfunctional connectivity was defined as a binary, as any value falling below a given value relative to the normative control sample would be marked as impaired.

This approach breaks assumptions of linear relationships between connections and phenotypes are complex behavior. Most importantly, by exploring the regional the network level, the authors are not assuming that the same set of connections will be impaired across all individuals with the given set of diagnosis, thus better accounting for individual variability and heterogeneity in clinical samples.

This approach has been used in prior work by the authors using different modalities, but represents a significant and novel break from standard analysis approaches.

Generally speaking, the approach is excellent, the work is topical, and it fits well with ongoing work from this research group. This approach provides opportunities to overcome challenges that have been noted in brainwide association studies, which assume common sets of impaired networks and linear relationships between FC or brain activity and complex behaviors or phenotypes. I also appreciate that results for higher and lower Z-threshold are presented, considerations of motion, and the sensitivity analyses.

I would consider this paper overall to be excellent. My primary concern relates to how the methods are presented. I appreciate the challenge of condensing such a study to the word count required by the journal, but generally speaking I found the methods extremely opaque. I was unable to understand specifically what was done in many cases, essentially having to make guesses or assumptions based on what I know from the author's prior work. I will lay out some specific cases where I feel the methods need to be clarified, but generally speaking, substantially more detail and clarification needs to be added to the methods, much of which could be potentially put in supplemental material.

The samples are well described, largely in the supplement, as is the imaging processing.

Response: We thank the reviewer for the positive feedback and appreciate the thoughtful comments on the strengths of our manuscript. In the following, we have addressed each of the Reviewer’s specific concerns.

Comment 2: The gaussian gamma mixture model approach could be briefly expanded upon, as many readers will not be familiar with this approach, this reviewer included.

Response: We appreciate the reviewer's suggestion to expand on the gaussian gamma mixture model. We have added this sentence on page 7 paragraph 1 of the revised manuscript:

> "This approach separates meaningful connectivity values from background noise by modelling their distinct statistical distributions, effectively suppressing connections likely to be noise."

We have also amended the Supplement section 5:

> **"**Section 5. Application of Mixture Modeling Normalization
> Mixture modeling normalization offers a statistically principled approach for addressing a fundamental challenge of functional connectivity studies: namely, that the precise threshold that should be used to distinguish meaningful from spurious correlation values is often unclear. The method works by decomposing the distribution of connectivity values into signal and noise components. After applying a Fisher-z transformation to the correlation values, a three-component mixture model is fitted: a central Gaussian distribution captures the bulk of near-zero values (interpreted as noise), while two Gamma distributions capture the positive and negative tails (interpreted as genuine signal). The Gamma distributions are thus used to capture the meaningful FC estimates (both positive or negative), which are relatively sparse and appear in the tails of the distribution of FC values.
> In a second step, each connectivity value across all three distributions is z-score normalized using the mean and standard deviation of the noise distribution. The resulting z-values quantify, in units of standard deviation, the degree to which a given FC estimate deviates from the noise floor. Connections hovering near zero remain near zero, while those representing true signal are amplified in proportion to their deviation from noise. In this way, the procedure acts as a soft threshold that enhances signal-to-noise ratio without imposing a hard cutoff that arbitrarily discards potentially meaningful connections. For further details, see (Chauvin et al., 2021; Chauvin, Mennes, Buitelaar, & Beckmann, 2018; Chauvin, Mennes, Llera, Buitelaar, & Beckmann, 2019; Alberto Llera, Huertas, Mir, & Beckmann, 2019; A Llera, Pruim, Wiegerinck, & Beckmann, 2015; A Llera, Vidaurre, Pruim, & Beckmann, 2016; Tyszka, Kennedy, Paul, & Adolphs, 2014)."

Comment 3: The tenfold cross validation used validate the model in the normative model section and supplemental section 7 is not well explained. I guess the r values in section 7 are the predicted versus actual on the left out fold? Please clarify. Relatedly Supp figure 3, graphs are not labeled. I can only assume the graphs are describing what is in the captions separated by semi-colons; this is not the ideal way to present data.

Response: We apologize for any confusion. To clarify the methodology: the 10-fold cross-validation is not a validation analysis, but rather the procedure through which we obtain normative deviations for the control group itself. Normative modeling requires that individuals

are evaluated against a model that did not include those same individuals when training the model. Therefore, to obtain the normative deviations for controls, we iteratively train on 9 folds and compute deviations for the held-out fold, cycling through until every control participant has been evaluated against a model fitted without their data. This yields unbiased deviation estimates for the entire control sample.
The model validation statistics are reported separately in Supplementary Section 7 to demonstrate that the normative models adequately capture the age, sex and mean framewise displacement-related variance in connectivity.
We have made the following changes.

First, we have revised our description of the cross-validation procedure as follows (page 7, paragraph 3 of the revised manuscript):

> “To obtain deviations for the group of autistic individuals, the model was trained on neurotypical and tested on autistic participants, establishing their deviations from the normative model. To obtain deviations for neurotypical individuals, we used 10-fold cross-validation, where we trained the model on 9 folds and tested it on the 10th held-out fold. This was repeated across all folds to obtain deviation estimates for the entire sample, whereby each control participant's deviations were computed from a model trained without their data. Separately, to assess whether the normative modelling procedure was successful, we computed normative model validation statistics which can be found in Supplement section 7.”

Second, we have added axis labels and amended the caption of Supplementary Figure S5 so that it is easier to interpret:

Redacted

Figure S5. Distributions of the normative modelling evaluation metrics for the group of neurotypical individuals.
Explained variance by the normative model across edges (i.e., the proportion of variance in the target variable that is predictable from the input features, with higher values

indicating better model fit); Mean Standardized Log Loss (MSLL) which compares the log loss of the model to that of a simple baseline predictor that always predicts the mean of the training data; Kurtosis and skew of the distribution of pseudo-z scores (values near 3 and near zero, respectively, indicate well-calibrated model estimates consistent with a standard normal distribution).

Comment 4: Notation $OR,G(\tau)$ is not well defined, and doesn't really add anything to the paper as it's never referred to again. Honestly, in this regional analysis section I really don't understand at all what's being done, or how or why there are thresholds. What is the threshold of zero mean? I can only guess what's actually being measured here, but I would have assumed that since the connections are defined as 0 or 1 for deviant or not, that it would simply be a count of how many connections from that region fell into the deviant range. Likewise I'm not sure what the area under the curve is measuring. This section is very unclear, I would have assumed it was total number of deviant connections per node but it looks like perhaps the number of people that have an exact match of which connections are deviant from which regions, Or a certain number of overlap? Very confused.

Same for the following section on network analysis, with 1 to 10%. It's not clear to me why overlap across participants would be more sensible than simply counting the connections, or how that overlap is actually calculated. Please clarify.

Figure 2 helps explain possibly some aspects of the methods, but it's still not clear why a threshold would be applied to count the number of participants, why this is called overlap, and what the AUC measures. Some of the confusion may stem from unclear use of the term 'overlap'.

Response: We thank the reviewer for drawing this to our attention. The challenge is that analyses at the connection level require a different analytic strategy to those at the region/network level. At the connection level, each connection is binary (deviant or not), so group comparisons are easy: we simply count how many participants show a deviation at each connection. At the region level, however, deviation degree (i.e., the number of deviant connections attached to a region) is not binary. For instance, one person may have a value of 10 in a given brain region and another person may have a value of 20. How do we define overlap in this case?

Our solution is to set a threshold, labeling an entire region as deviant if its deviation degree exceeds some number. But of course, this raises the question of how such a threshold should be chosen. Rather than rely on a single arbitrary choice, we therefore evaluated group differences across a range of thresholds (1 to 20). The data can then be plotted as a 2D curve with threshold on the x-axis and the number of participants showing a deviation degree above the threshold (our measure of ‘overlap’) on the y-axis (as shown in Fig 2). We then summarize the findings across all thresholds using the area under the curve (AUC). A region showing high levels of overlap will do so across a range of thresholds, yielding a higher AUC. Our approach

thus provides a threshold-free summary measure that can be compared between groups using permutation testing.

We have amended page 8, paragraph 2 of the revised the manuscript to clarify our approach:

> "For each participant, we counted the number of connections with extreme z-scores attached to each brain region (|z| > 2.3; Figure 2B), a quantity we term deviation degree. We then used deviation degree to study the level of deviation overlap at the regional level. At the connection level, each connection, an FC deviation estimate can be classified as deviant or not depending on whether it exceeds the threshold. Computing overlaps across participants in this scenario is straightforward. However, at the region level, , deviation degree is not binary (e.g., a region might have 0, 3, or 12 deviant connections), complicating attempts to quantify overlap across participants. One approach would be to apply a threshold to deviation degree values, but the specific threshold value that should be used is unclear. We therefore evaluated group differences across a range of thresholds, $1 < \tau < 20$, and, at each threshold, plotted how many participants had a deviation degree of at least τ (Figure 3B). The upper bound of 20 was chosen because few people showed higher deviation degree within any brain region. We then calculated the area under the curve (AUC) across these thresholds, providing a single summary measure that captures regional overlap across groups without depending on any particular threshold choice. We compared AUC values between autistic and control groups using 10,000 permutations of diagnostic labels, and used FDR-correction across all regions."

Comment 5: The results are highly compelling and generally well presented, saving that they're hard to contextualize because the methods are unclear.

Response: We thank the reviewer for their positive assessment of our results. We hope that the revisions above provide sufficient clarity and context.

Comment 6: Figure 3 D caption And graph labels, 'relative differences' should be clarified, e.g. Is it the percent AD minus Percent NT?

Response: Because the AUC is a summary measure with no intuitive scale, we report group differences as relative differences:

$$\text{Relative difference} = \frac{\text{AUC}_{\text{autism}} - \text{AUC}_{\text{neutotypical}}}{\text{AUC}_{\text{neurotypical}}}.$$

This quantity expresses the autism group's AUC as a proportional change relative to the control group. For example, a value of 0.15 indicates that the autism group's AUC is 15% larger than controls, while −0.10 would indicate it is 10% smaller. This makes effect sizes interpretable and comparable across regions and networks.

We have added the following text to the caption of Figure 3:

> "Between-group differences are presented as the percentage difference in the overlap between the groups i.e. $\text{Relative difference} = \frac{\text{AUC}_{\text{autism}} - \text{AUC}_{\text{neutotypical}}}{\text{AUC}_{\text{neurotypical}}}$. This measure represents the proportional difference in area under the curve between groups."

Comment 7: The behavioral predictions analysis was quite interesting, and highlights how different levels of analysis may map on the different behavioral findings. I enjoyed the discussion of this as well.

Response: We thank the reviewer for this positive feedback.

Comment 8: The discussion is generally very good, though it relies a lot on a small number of prior papers from this group. It might be better contextualized in the broader literature, for example these results fit quite well with the lesion network model championed by these results fit quite well with the lesion network modelling approach championed by Fox and Siddiqi, showing that disparate regional findings can map onto a common Network.

Response: We thank the reviewer for the positive feedback on our discussion and on highlighting the connection and alignment of our finding with the lesion network modelling approach by Fox and Siddiqi. We added the following text to our discussion, page 15:

> "Our finding that heterogeneous connection-level deviations converge onto common regions and networks aligns with lesion network mapping studies showing that brain lesions causing neuropsychiatric symptoms, despite heterogeneous locations, map onto shared functional circuits when projected onto normative connectivity data (Fox, 2018) (Shan H Siddiqi, Kording, Parvizi, & Fox, 2022). Siddiqi and colleagues demonstrated this principle for depression, where both lesions and therapeutic stimulation sites converged on a common circuit (Padmanabhan et al., 2019; Shan H. Siddiqi et al., 2021). Our results extend this framework to neurodevelopmental conditions. Specifically, we find that while no single connection reliably distinguishes autistic from neurotypical individuals, FC deviations preferentially aggregate within specific networks or attach to specific regions. "

Comment 9: The only potential additional analysis I might like to see is some sort of estimate of stability or overlap of findings if the data was sub-sampled. The sensitivity analysis across data sets helped a lot in this regard, but it would be interesting to see if various splits of the data showed similar patterns in the regional and Network levels. This is of potential import given the heterogeneity seen across studies, which this paper is attempting to address.

Response: To evaluate the stability of our network- and region-level findings, we performed bootstrap resampling analyses (n = 1,000 iterations with replacement). For each iteration, we resampled subjects independently within the autistic and neurotypical groups, maintaining original sample sizes, and recomputed all overlap metrics. We then assessed stability using two complementary approaches: (1) inter-bootstrap correlations, quantifying the similarity of effect patterns across resampled datasets; and (2) direction consistency, measuring how often each feature showed the same direction of group difference as the primary analysis. This bootstrapping procedure is a widely-accepeted statistical approach for quantifying the stability of one's results.

We now report the results in the supplement Section 12 and Supplementary Figure S9 of the revised manuscript, as detailed below. The analysis confirms that our findings are robust to sampling variability and that they are not driven by influential observations or idiosyncratic sample characteristics.

> "To further assess the stability of our findings, we performed a bootstrap reliability analysis with 1,000 iterations, resampling participants with replacement within each group. For each iteration, we recomputed group difference maps and evaluated their consistency with the main results. At the network level, inter-bootstrap correlations indicated good reliability for positive deviations (mean r = 0.814) and acceptable reliability for negative deviations (mean r = 0.733). Region-level analyses showed a similar pattern (positive deviations mean r = 0.829; negative deviations mean r = 0.663). Analysis of the consistency of the direction of the group differences further confirmed that most network pairs and regions maintained the same effect polarity across bootstrap samples, with mean consistency exceeding 88% for both positive and negative deviations at the region level, and with 100% consistency in significant networks (Figure S9). Together, these results indicate that both the magnitude and direction of our reported group differences are robust to sampling variability."

Redacted

Figure S9. Bootstrap reliability analysis of network- and region-level deviation overlap. A) Distribution of inter-bootstrap correlations for network-level analyses from 1,000 bootstrap iterations. Dashed lines indicate mean and median; dotted lines indicate 95% confidence intervals. B) Direction consistency matrices showing the percentage of bootstrap samples in which each network pair exhibited the same direction of effect (ASD > TD or TD > ASD) as the observed data. Values represent consistency percentages across 1,000 bootstrap iterations. Most network pairs showed high consistency (>80%), indicating stable effect directions. C) Distribution of inter-bootstrap correlations for region-level analyses (390 regions).

Comment 10: Overall an excellent paper. The conclusions are appropriate to the data, the use of statistics appears robust, and the work is timely given awareness of issues and heterogeneity and challenges that the brainwide associations observed at the connection level.

Once again, we thank the Reviewer for their positive appraisal.

References:

Chauvin, R. J., Buitelaar, J. K., Sprooten, E., Oldehinkel, M., Franke, B., Hartman, C., . . . Beckmann, C. F. (2021). Task-generic and task-specific connectivity modulations in the ADHD brain: an integrated analysis across multiple tasks. *Translational psychiatry, 11*(1), 1-10.

Chauvin, R. J., Mennes, M., Buitelaar, J. K., & Beckmann, C. F. (2018). Assessing age-dependent multi-task functional co-activation changes using measures of task-potency. *Developmental Cognitive Neuroscience, 33*, 5-16.

Chauvin, R. J., Mennes, M., Llera, A., Buitelaar, J. K., & Beckmann, C. F. (2019). Disentangling common from specific processing across tasks using task potency. *Neuroimage, 184*, 632-645.

Fox, M. D. (2018). Mapping symptoms to brain networks with the human connectome. *New England Journal of Medicine, 379*(23), 2237-2245.

Llera, A., Huertas, I., Mir, P., & Beckmann, C. F. (2019). Quantitative intensity harmonization of dopamine transporter SPECT images using gamma mixture models. *Molecular imaging and biology, 21*(2), 339-347.

Llera, A., Pruim, R., Wiegerinck, W., & Beckmann, C. (2015). *Gaussian/Inverse Gamma mixture models of ICA maps.* Paper presented at the 21th Int. Conf. on Functional Mapping of the Human Brain.

Llera, A., Vidaurre, D., Pruim, R., & Beckmann, C. (2016). Variational mixture models with gamma or inverse-gamma components. *arXiv preprint arXiv:1607.07573*.

Padmanabhan, J. L., Cooke, D., Joutsa, J., Siddiqi, S. H., Ferguson, M., Darby, R. R., . . . Voss, J. L. (2019). A human depression circuit derived from focal brain lesions. *Biological Psychiatry, 86*(10), 749-758.

Siddiqi, S. H., Kording, K. P., Parvizi, J., & Fox, M. D. (2022). Causal mapping of human brain function. *Nature reviews neuroscience, 23*(6), 361-375.

Siddiqi, S. H., Schaper, F. L. W. V. J., Horn, A., Hsu, J., Padmanabhan, J. L., Brodtmann, A., . . . Fox, M. D. (2021). Brain stimulation and brain lesions converge on common causal circuits in neuropsychiatric disease. *Nature Human Behaviour, 5*(12), 1707-1716. doi:10.1038/s41562-021-01161-1

Tyszka, J. M., Kennedy, D. P., Paul, L. K., & Adolphs, R. (2014). Largely typical patterns of resting-state functional connectivity in high-functioning adults with autism. *Cerebral cortex, 24*(7), 1894-1905.

Reviewer #2:

Comment 1: This manuscript investigates the critical challenge of inter-individual heterogeneity in autism by examining atypical functional connectivity (FC) across multiple scales of brain organization. Utilizing a large, multi-site resting-state fMRI dataset (N=1824) from the EU-AIMS LEAP and ABIDE consortia, the authors employ normative modeling to quantify individual deviations from expected FC patterns, controlling for age, sex, and head motion.
The study reveals that FC heterogeneity in autism is scale-dependent. At the level of individual connections, deviations were highly idiosyncratic, with minimal overlap across participants (<4%), and the overall burden of deviations was similar between the autistic and neurotypical (NT) groups. However, at coarser scales (regions and networks), deviations in the autistic group showed significantly higher convergence. Specifically, the results highlight convergent hypoconnectivity (negative deviations) within sensorimotor and attention systems, and hyperconnectivity (positive deviations) involving the frontoparietal and default mode networks. These deviation patterns showed modest but statistically significant associations with clinical and cognitive abilities.
This is an excellent study characterized by exceptionally neat technical practices and sophisticated analytical methods. The authors are to be congratulated on this impressive work. The utilization of a large aggregated dataset, rigorous preprocessing, careful harmonization across 32 sites, and the implementation of Gaussian Process Regression (GPR) for normative modeling are highly laudable. The multiscale approach offers a compelling neurobiological framework for reconciling shared diagnostic criteria with extensive phenotypic variability, moving the field beyond the limitations of traditional case-control comparisons.
I support its publication pending revisions addressing the comments detailed below, which aim to enhance the clarity, robustness, and interpretation of these important findings.

Response: We thank the reviewer for their thorough summary of our work, and for their positive assessment of our analytical approach. We appreciate the support for publication and have carefully addressed each specific comment below.

Major Comments
Comment 2: The concept of "multiscale" analysis is central to the manuscript. While the approach is visualized (Figures 1 and 2), the theoretical motivation and rationale for specific methods could be articulated more clearly, particularly for a broad audience that includes clinicians. Please expand the Introduction to better elaborate on the rationale for expecting differences in heterogeneity across scales (connection, region, network). Why might heterogeneity decrease as the spatial scale coarsens? Articulating the theoretical underpinnings (e.g., diverse etiological pathways converging on common functional systems) will help readers interpret the significance of the observed scale-dependent heterogeneity.

Response: We thank the reviewer for this suggestion. We have expanded the Introduction to articulate the theoretical rationale for expecting decreased heterogeneity at coarser spatial scales.

The principle that diverse disruptions converge at higher organizational levels is well-established in autism genetics itself. Despite over 100 identified risk genes, each affecting different proteins and molecular mechanisms, these genes converge on a limited set of biological pathways, particularly synaptic function and transcriptional regulation (De Rubeis et al., 2014; Pinto et al., 2014; Satterstrom et al., 2020). We propose that this same principle extends to brain connectivity: just as genetically heterogeneous mutations converge on shared molecular pathways, individually variable connection-level disruptions may converge on shared brain regions and networks. Segal et al. (2023) recently demonstrated this for gray matter volume across psychiatric disorders, finding that highly heterogeneous regional deviations were embedded within common functional circuits in up to 56% of cases. We added this paragraph to the introduction, page 4:

> "The brain is organized hierarchically, such that specific inter-regional connections are embedded within regions, which belong to broader functional networks. As such, it is possible that different autistic individuals may display disruptions of distinct connections, but that these disrupted connections may nonetheless be concentrated on or within specific regions or networks. A similar principle has been established in autism genetics, where over 100 identified risk genes linked to distinct molecular mechanisms nonetheless converge on a limited set of biological pathways related, in particular, to synaptic function and transcriptional regulation (De Rubeis et al., 2014; Pinto et al., 2014; Satterstrom et al., 2020). We propose that this convergence extends to brain organization, such that variable connection-level disruptions may converge on shared regions and networks. Segal et al. (2023) recently demonstrated this phenomenon for person-specific deviations of gray matter volume across psychiatric disorders. Here, we test whether the same scale-dependent convergence characterizes atypical FC in autism."

Comment 3: Further, please provide brief justifications in the main text Methods section for key analytical decisions. For instance (not limited to), provide a brief rationale for the use of Gaussian-gamma mixture modeling (e.g., its advantage in enhancing differentiation of signal from noise). Also, please clarify the rationale for the specific choice of $|Z|>2.3$ ($p=0.01$) as the primary threshold for extreme deviations.

Response: We have made two modifications to the revised manuscript to clarify our Gaussian mixture modelling procedure.

First, on page 7, paragraph 1, we write:

> “This approach separates meaningful connectivity values from background noise by modelling their distinct statistical distributions, effectively suppressing connections likely to be noise.”

Second, in section 5 of the Supplementary Material we write:

“Section 5. Application of Mixture Modeling Normalization
Mixture modeling normalization offers a statistically principled approach for addressing a fundamental challenge of functional connectivity studies: namely, that the precise threshold that should be used to distinguish meaningful from spurious correlation values is often unclear. The method works by decomposing the distribution of connectivity values into signal and noise components. After applying a Fisher-z transformation to the correlation values, a three-component mixture model is fitted: a central Gaussian distribution captures the bulk of near-zero values (interpreted as noise), while two Gamma distributions capture the positive and negative tails (interpreted as genuine signal). The Gamma distributions are thus used to capture the meaningful FC estimates (both positive or negative), which are relatively sparse and appear in the tails of the distribution of FC values.
In a second step, each connectivity value across all three distributions is z-score normalized using the mean and standard deviation of the noise distribution. The resulting z-values quantify, in units of standard deviation, the degree to which a given FC estimate deviates from the noise floor. Connections hovering near zero remain near zero, while those representing true signal are amplified in proportion to their deviation from noise. In this way, the procedure acts as a soft threshold that enhances signal-to-noise ratio without imposing a hard cutoff that arbitrarily discards potentially meaningful connections. For further details, see (Chauvin et al., 2021; Chauvin, Mennes, Buitelaar, & Beckmann, 2018; Chauvin, Mennes, Llera, Buitelaar, & Beckmann, 2019; Alberto Llera, Huertas, Mir, & Beckmann, 2019; A Llera, Pruim, Wiegerinck, & Beckmann, 2015; A Llera, Vidaurre, Pruim, & Beckmann, 2016; Tyszka, Kennedy, Paul, & Adolphs, 2014).”

Regarding the choice of threshold of z=2.3, we added the following to pages 7 and 8 of the Methods section:

“Extreme deviations were defined as $|Z| > 2.3$, corresponding to approximately $p < 0.01$. Thresholds for defining extreme deviations in normative modeling studies have typically ranged from $|Z| > 1.96$ ($p < 0.05$)(Bayer et al., 2022; Lv et al., 2021) to $|Z| > 2.6$ ($p < 0.005$) (Liu et al., 2025; Sun et al., 2023; Wolfers et al., 2020). The present threshold represents a principled intermediate choice that balances sensitivity to meaningful deviations against specificity. Given that a central aim of this study is to characterize heterogeneity in autism and capture the diversity of individual-level atypical connectivity patterns, we favoured a threshold that maintains sensitivity rather than a more stringent cutoff that risks obscuring genuine variability. To ensure robustness, we report supplementary analyses at $|Z| > 1.96$ ($p < 0.05$), $|Z| > 2.6$ ($p < 0.005$), and $|Z| > 3.1$ ($p < 0.001$), which demonstrate consistent spatial patterns with the expected attenuation at more extreme thresholds (Supplement Section 9, Figures S6 and S7).”

Comment 4: The analyses are based on the Schaefer 400-region cortical parcellation (plus

subcortical ROIs). As the definition of nodes in connectomics inherently influences the resulting metrics, it is crucial to demonstrate that the findings are robust to the specific resolution of the chosen parcellation. The authors should repeat the main overlap analysis (reported in Figure 3) using different parcellation resolutions (e.g., a coarser Schaefer 200 and a finer Schaefer 800 atlas).

Response: We have now repeated the main overlap analysis (Figure 3) using both a coarser (Schaefer 200) and a finer (Schaefer 800) parcellation. The results are consistent across resolutions, with the spatial patterns of group-level overlap in both positive and negative deviations showing the same overall profile as reported with the Schaefer 400 parcellation. Our conclusions remain unchanged.

We report these additional analyses in the Supplement (Figure S8), and add the following text:

> "To assess whether our findings are robust to the choice of parcellation resolution, we repeated the main overlap analysis using both a coarser (Schaefer 200-region) and a finer (Schaefer 800-region) cortical parcellation. The spatial distribution of group differences in both positive and negative deviations was consistent across resolutions, reproducing the patterns observed in the primary Schaefer 400 analysis (Figure S8). This confirms that our results are not dependent on the granularity of the parcellation."

Redacted

Figure S8. Parcellation resolution sensitivity analysis. Overlap of extreme deviations between autistic and neurotypical groups repeated using the Schaefer 200-region (left) and Schaefer 800-region (right) cortical parcellations. Top rows (green) show negative deviations; bottom rows (purple) show positive deviations. For each parcellation, cortical surface maps display the relative significant difference of regional, and matrices show the relative difference in overlap between groups at the network level.

Comment 5: The authors include sex as a covariate in the GPR model. This approach typically

models sex as an additive effect, assuming that the trajectory of FC across age is parallel between males and females. Given the established sex differences in neurodevelopmental trajectories and autism, this assumption may not hold. Modeling sex merely as a covariate, without accounting for interactions (e.g., age-by-sex), can lead to inaccurate norms and potentially bias the resulting deviation scores. It is recommended to formally test for age-by-sex interactions. If significant interactions are present, or if model fit differs substantially between sexes, the normative models should ideally be stratified (trained separately for males and females) to ensure accurate modeling, although the constraints of the female sample size are acknowledged.

Response: We thank the reviewer for raising this important point. Following the reviewer's suggestion, we tested the data pre normative modelling for age-by-sex interactions across all 75,855 functional connections by fitting a linear model at each connection with age, sex, mean framewise displacement, and an age-by-sex interaction term. After FDR correction (Benjamini-Hochberg, $q < 0.05$), only 2 out of 75,855 connections in the pre-normative modelling data showed a significant interaction, suggesting that age-related trajectories of FC are largely similar between males and females in our sample. These results provide empirical support for the additive modelling of sex in our GPR normative models. We added the following paragraph to the Supplement :

> "We additionally tested for age-by-sex interactions on pre-normative modelling FC estimates by fitting a linear model at each connection with age, sex and mean framewise displacement across all 75,855 connections. The analysis revealed only 2 significant interactions after FDR correction ($q < 0.05$), lending support for our use of an additive age, sex and mean framewise displacement model."

Comment 6: The study covers a very wide age range (5-58 years). Normative models are typically least stable and most uncertain at the extremes of the covariate distribution where data may be sparse. To assess the reliability of the model across the lifespan, please provide a visualization (e.g., histogram or density plot) of the age distribution for the training sample (NT controls). Please visualize or discuss the uncertainty of the predictions (e.g., the predictive variance from the GPR) across the age range, particularly at the younger and older ends.

Response: We agree that examining the distribution of the training data and associated prediction uncertainty is essential for interpreting normative modeling results. As requested, we now provide a histogram of the age distribution for the neurotypical training sample (Figure S10 of the revised manuscript). The distribution shows that the majority of participants fall within the 6–30 year age range, with peak density between approximately 10–20 years. Data become progressively sparser beyond 35 years, with very few participants over 45 years.

Redacted

Figure S10. Age distribution of the group of neurotypical individuals
The findings are most robust within the 8–35 year range, where we have the most coverage in our training data. This range covers 93% of autistic individuals in our sample.

The GPR framework appropriately propagates uncertainty and predictive variance increases in data-sparse regions by yielding more conservative z-scores at the age extremes. While this reduces false positive risk, it also limits statistical power to detect true deviations in these regions. The findings are therefore most robust in the well-sampled 8–35 year range, which covers the age range of 93% of our autistic sample. We now acknowledge these issues on page 17, in the limitations section of the revised manuscript:

> "Our ability to detect deviations is reduced at the extremes of the age distribution (below 8 and above 35 years), where sparser training data lead to increased predictive uncertainty. The findings are most robust within the 8–35 year range, where we have the most coverage in our training data. This range covers 93% of autistic individuals in our sample."

Comment 7: As detailed in the Supplement (Section 2), participants with IQ < 70 were excluded. Table 1 confirms the resulting sample is high-functioning (Mean IQ Autism ≈ 106). This significantly limits the generalizability of the findings to the entire autism spectrum, which includes individuals with intellectual disabilities. This limitation must be explicitly addressed in the main text's Limitations section, and the conclusions should reflect the specific population studied.

Response: We agree. We have added an explicit acknowledgement to the Limitations section that our findings are based on a sample without intellectual disability and may not generalise to

the full autism spectrum, including individuals with co-occurring intellectual disability. We have also adjusted the conclusions to reflect this.

> "Our analysis only included individuals without intellectual disability (IQ ≥ 70). As a result, our findings may not generalise to autistic individuals with co-occurring intellectual disability."

Comment 8: The multivariate predictive models (Figure 4) show statistically significant correlations, but the effect sizes are modest (median correlations ranging from 0.1 to 0.29). The discussion and abstract (e.g., "representing viable targets for biomarker... development") should be tempered to reflect the modest strength of these associations and the challenges remaining before clinical translation.

Response: We have now tempered the last sentence of the abstract and we do not mention biomarker potential in the discussion.

> "These findings demonstrate that autism exhibits scale-dependent heterogeneity, characterized by normative variability at the connection level but significant convergence at regional and network scales. These convergent regions and networks may be used to identify targets for individualized therapeutic development."

Comment 9: The authors conclude that connection-level heterogeneity is within "normative expectations" because the overlap in the autistic group is similar to the control group. However, the estimation procedures differ: control deviations were quantified using 10-fold cross-validation (within the training data), whereas autism deviations were quantified by applying the NT-trained model to the held-out autism data. The authors should discuss the nuances of comparing deviations derived from a test set (autism) versus a cross-validated training set (controls) when interpreting the baseline level of heterogeneity.

Response: We clarify that while the estimation procedures do differ in implementation, they are designed to achieve the same goal: obtaining unbiased, out-of-sample deviation estimates for each individual. Specifically, the 10-fold cross-validation procedure ensures that each control participant's deviations are derived from a model trained without their own data, meaning that, as with the autistic participants, no individual's deviation scores are biased by their contribution to model training. In both cases, deviations are therefore estimated in an out-of-sample manner, and this makes the resulting scores comparable across groups. We have added an improved explanation to the manuscript, page 7, to clarify this aspect of the approach.

> "To obtain deviations for the group of autistic individuals, the model was trained on neurotypical and tested on autistic participants, establishing their deviations from the normative model. To obtain deviations for neurotypical individuals, we used 10-fold cross-validation, where we trained the model on 9 folds and tested it on the 10th held-out fold. This was repeated across all folds to obtain deviation estimates for the entire

sample, whereby each control participant's deviations were computed from a model trained without their data.”

Comment 10: Table 1 indicates a significant difference in mean FD between the groups ($p < .0001$). While the authors employed rigorous denoising (validated in Supplement Section 4) and included mean FD in the normative model, two concerns remain. First, to confirm that motion effects were successfully modeled as a covariate, please report the correlation between the final deviation scores (Z-scores) or the total number of extreme deviations per participant and mean FD for both groups. Second, given that the findings highlight significant convergence in the sensorimotor network—an area susceptible to motion artifacts—the authors should briefly discuss the spatial topography of their findings and argue why these results are likely neurobiological rather than residual artifacts.

Response: To address the first point, we computed the correlation between mean FD and the deviation scores (Z-scores) for every connection in both groups using both standard correlations and partial correlations controlling for age and sex. No significant correlations were observed after correction for multiple comparisons, indicating that residual motion effects are not systematically driving the deviation scores. This result also indicates that head motion cannot explain our findings within sensorimotor areas. We have added these results to the Supplement.

> “Additionally, we tested the correlation between mean FD and the deviation scores (Z-scores) for every connection in both groups. No significant correlations were observed after correction for multiple comparisons, even when partialling the effects of age and sex, indicating that residual motion effects do not systematically driving the deviation scores. ”

We have also added the following to page 17, paragraph 2 of the revised manuscript:

> “We observed group differences in head motion, as quantified using mean FD, which may raise concerns about our findings, particularly those within somatomotor areas. However, we found no significant correlations between deviation scores and mean FD, suggesting that residual motion effects are unlikely to explain our findings.”

Minor Comments

Comment 11: For a multi-site study of this scale, please provide diagnostics showing the efficacy of ComBat harmonization (e.g., variance explained by site before and after harmonization) in the Supplement.

Response: We now include a supplementary figure (Figure S4) demonstrating the efficacy of the ComBat harmonization. This figure shows that prior to harmonization, the majority of functional connectivity edges showed significant site effects ($p < 0.05$) and scanning sites were

readily classifiable from the connectivity data using a linear SVM (AUC ROC well above chance). After ComBat, significant site effects were virtually eliminated and classification performance dropped to near chance across all site pairs. We have added a paragraph to Supplement Section 6 describing these results.

> “Figure S4 demonstrates the effectiveness of the ComBat harmonization procedure on our data. Prior to harmonization, most FC estimates showed significant site effects ($p < 0.05$) and a linear SVM classifier could readily distinguish scanning sites from the connectivity data, with AUC ROC scores well above chance for nearly all site pairs. After ComBat, significant site effects were virtually eliminated across all connections, and classification performance dropped to near chance levels, confirming that site-related variance was effectively removed while preserving biologically relevant signal”

Redacted

Figure S4. Evaluation of scanning site effects before and after ComBat harmonization. Top panels show functional connectivity edges with significant (yellow, $p < 0.05$) and non-significant (blue, $p > 0.05$) site effects, tested for each pairwise connection. Before ComBat (left), the majority of connections show significant site effects; after ComBat (right), virtually none remain. Bottom panel shows area under the receiver operating characteristic curve (AUC ROC) scores from linear SVM one-vs-one classification of scanning sites. Before harmonization (blue), sites are readily distinguishable from the connectivity data (AUC ROC well above 0.5). After ComBat (red), classification performance drops to near chance across all site pairs, confirming effective removal of site-related variance.

Comment 12: Reference 10 (Segal A, Parkes L, Aquino K, et al. medRxiv. 2022) has been published. Please update the citation.

Comment 13: Please check for citation consistency throughout the text. There are frequent instances of spaces between the text and the citation.

Comment 14: Line 176: "...with the upper bound of 10% chose because..." should be "chosen".

Comment 1:5 There is a discrepancy in the final sample sizes reported. The main text reports 796 autistic and 1028 NT individuals. The Supplement (Section 3, p. 4, last paragraph) reports 773 autistic individuals and 994 NT individuals. Please clarify this discrepancy.

Comment 16: Please review the supplements for minor typos.

Response: We thank the reviewer for noticing and pointing out these minor comments. We have carefully addressed them all in the manuscript and supplement accordingly.

References:

Bayer, J. M. M., Dinga, R., Kia, S. M., Kottaram, A. R., Wolfers, T., Lv, J., . . . Marquand, A. (2022). Accommodating site variation in neuroimaging data using normative and hierarchical Bayesian models. *Neuroimage, 264*, 119699. doi:10.1016/j.neuroimage.2022.119699

Chauvin, R. J., Buitelaar, J. K., Sprooten, E., Oldehinkel, M., Franke, B., Hartman, C., . . . Beckmann, C. F. (2021). Task-generic and task-specific connectivity modulations in the ADHD brain: an integrated analysis across multiple tasks. *Translational psychiatry, 11*(1), 1-10.

Chauvin, R. J., Mennes, M., Buitelaar, J. K., & Beckmann, C. F. (2018). Assessing age-dependent multi-task functional co-activation changes using measures of task-potency. *Developmental Cognitive Neuroscience, 33*, 5-16.

Chauvin, R. J., Mennes, M., Llera, A., Buitelaar, J. K., & Beckmann, C. F. (2019). Disentangling common from specific processing across tasks using task potency. *Neuroimage, 184*, 632-645.

De Rubeis, S., He, X., Goldberg, A. P., Poultney, C. S., Samocha, K., Ercument Cicek, A., . . . The Autism Sequencing, C. (2014). Synaptic, transcriptional and chromatin genes disrupted in autism. *Nature, 515*(7526), 209-215. doi:10.1038/nature13772

Liu, Q., Lai, H., Le, J., Lan, C., Zhang, X., Huang, L., . . . Zhao, W. (2025). Identifying brain functional subtypes and corresponding task performance profiles in autism spectrum disorder. *Molecular psychiatry, 30*(11), 5034-5044. doi:10.1038/s41380-025-03086-x

Llera, A., Huertas, I., Mir, P., & Beckmann, C. F. (2019). Quantitative intensity harmonization of dopamine transporter SPECT images using gamma mixture models. *Molecular imaging and biology, 21*(2), 339-347.

Llera, A., Pruim, R., Wiegerinck, W., & Beckmann, C. (2015). *Gaussian/Inverse Gamma mixture models of ICA maps.* Paper presented at the 21th Int. Conf. on Functional Mapping of the Human Brain.

Llera, A., Vidaurre, D., Pruim, R., & Beckmann, C. (2016). Variational mixture models with gamma or inverse-gamma components. *arXiv preprint arXiv:1607.07573*.

Lv, J., Di Biase, M., Cash, R. F. H., Cocchi, L., Cropley, V. L., Klauser, P., . . . Zalesky, A. (2021). Individual deviations from normative models of brain structure in a large cross-sectional schizophrenia cohort. *Molecular psychiatry, 26*(7), 3512-3523. doi:10.1038/s41380-020-00882-5

Pinto, D., Delaby, E., Merico, D., Barbosa, M., Merikangas, A., Klei, L., . . . Scherer, S. W. (2014). Convergence of genes and cellular pathways dysregulated in autism spectrum disorders. *Am J Hum Genet, 94*(5), 677-694. doi:10.1016/j.ajhg.2014.03.018

Satterstrom, F. K., Kosmicki, J. A., Wang, J., Breen, M. S., De Rubeis, S., An, J. Y., . . . Buxbaum, J. D. (2020). Large-Scale Exome Sequencing Study Implicates Both Developmental and Functional Changes in the Neurobiology of Autism. *Cell, 180*(3), 568-584.e523. doi:10.1016/j.cell.2019.12.036

Segal, A., Parkes, L., Aquino, K., Kia, S. M., Wolfers, T., Franke, B., . . . Fornito, A. (2023). Regional, circuit and network heterogeneity of brain abnormalities in psychiatric disorders. *Nature neuroscience, 26*(9), 1613-1629. doi:10.1038/s41593-023-01404-6

Sun, X., Sun, J., Lu, X., Dong, Q., Zhang, L., Wang, W., . . . Xia, M. (2023). Mapping Neurophysiological Subtypes of Major Depressive Disorder Using Normative Models of the Functional Connectome. *Biol Psychiatry, 94*(12), 936-947. doi:10.1016/j.biopsych.2023.05.021

Tyszka, J. M., Kennedy, D. P., Paul, L. K., & Adolphs, R. (2014). Largely typical patterns of resting-state functional connectivity in high-functioning adults with autism. *Cerebral cortex, 24*(7), 1894-1905.

Wolfers, T., Beckmann, C. F., Hoogman, M., Buitelaar, J. K., Franke, B., & Marquand, A. F. (2020). Individual differences v. the average patient: mapping the heterogeneity in ADHD using normative models. *Psychological Medicine, 50*(2), 314-323.

Reviewer #3:

Comment 1: While the present study applied normative modeling to quantify heterogeneity in functional connectivity in ASD, the methodological approach was not novel(DOI: 10.1038/s41380-025-03086-x; DOI: 10.1016/j.biopsych.2023.05.021.). The current findings just established patterns of heterogeneity without providing substantial new evidence or a novel theoretical perspective.

Response: We thank the reviewer for these references and for the opportunity to clarify how our work relates to this literature. Apologies for missing to reference them in our submission. The first cited study (Liu et al., 2025) uses normative modeling to identify discrete ASD subtypes by clustering deviation scores, integrating static and dynamic FC and relating the clusters with eye-tracking data. We see our work as complementary rather than redundant. Where Liu et al. focus on subtyping, our study addresses a different question: how FC heterogeneity is spatially organized across multiple levels of granularity, from individual connections, through regions, to functional networks.

The second cited study (DOI: 10.1016/j.biopsych.2023.05.021) applies clustering on normative modeling of functional connectivity measured in major depressive disorder. While the analytical framework uses normative modelling to derive subtypes, the clinical population and research questions differ from the present work.

The main novelty of our contribution is our multiscale perspective, which has not been considered in the literature. Our analysis reveals, that deviations occur in highly idiosyncratic ways at the connection level, but that these deviations nonetheless converge in meaningful ways at regional and network levels. This result provides a putative neural correlate of both the high clinical variability of autistic individuals (i.e., high connection-level heterogeneity) as well as of phenotypic similarities between such individuals (i.e., the emergence of canonical symptoms that result in a common diagnosis). High connection-level heterogeneity also explains why FC studies in the literature have reported such variable results, with both increases and decreases of FC being observed.

We clarify the novelty of our findings on page 17 and cite the work that the reviewer suggests in our response to Comment 2:

> “In summary, our results highlight the importance of adopting a multiscale approach to characterizing the heterogeneity of neural phenotypes in autism. This multiscale perspective reveals a novel organizational principle: while deviations at the level of specific connections are highly idiosyncratic, they converge into more consistent patterns at regional and network levels, offering a parsimonious account of how a common diagnosis might arise despite pronounced individual differences in underlying connectivity. Connection-level heterogeneity offers a plausible neural substrate for individual phenotypic differences and may explain the inconsistent FC findings reported in the literature thus far, where both increases and decreases have been observed across studies. Our findings further suggest that reduced FC of sensorimotor systems

and increased FC of transmodal association networks potentially reflect imbalanced signaling along the sensorimotor-association axis of the brain. FC deviations at distinct levels predict different clinical phenotypes, emphasizing the importance of considering multiple levels when characterizing brain-behavior relationships. These results replicate across datasets, different granularity of brain parcellations and multiple sensitivity analyses for individuals without intellectual disability.”

Comment 2: Lines 91-93: Normative modeling has revealed not only structure but function deviations. Please add more details. For example, DOI: 10.1038/s41380-025-03086-x; DOI: 10.1016/j.biopsych.2023.05.021.

Response: We now cite the two suggested manuscripts:

> “This approach has revealed highly individualized patterns of brain structure and function deviations across psychiatric conditions, suggesting that group averages poorly represent individual patient profiles(Liu et al., 2025; Marquand et al., 2019; Segal et al., 2023; Sun et al., 2023; Zabihi et al., 2019).”

Comment 3: Line 95: Please specific multiscale normative modeling in the introduction section.

Response: We now clarify this sentence in the introduction:

> “We applied multiscale normative modeling across three spatial scales, inter-regional connections, individual brain regions, and extended brain networks, to characterize the inter-individual heterogeneity of functional connectivity (FC), defined as inter-regional correlations in resting-state functional magnetic resonance imaging (fMRI) signals, in a large multisite dataset of people with autism.”

Comment 4: Line 153,163: In contrast to previous studies that used a stringent threshold (e.g., Z = ±2.58, 2.6) for deviation maps (Sun et al., 2023; Liu et al., 2025; Wolfers et al., 2018; Wolfers et al., 2020), the current study employed a more liberal threshold of Z = ±2.3. This less conservative approach captures a broader range of deviations. Ref:https://doi.org/10.1001/ jamapsychiatry.2018.2467;https://doi.org/ 10.1017/S003329171900 0084.

Response: A central aim of our study is to characterize heterogeneity in autism, capturing the diversity of atypical connectivity patterns across individuals. A lenient threshold will result in too much overlap and an overly stringent threshold will reduce sensitivity to the inherent heterogeneity of the diagnosis by excluding meaningful individual-level deviations. Thresholds for defining extreme deviations in normative modeling studies have typically ranged from $|Z| > 1.96$ ($p < 0.05$)(Bayer et al., 2022; Lv et al., 2021) to $|Z| > 2.6$ ($p < 0.005$) (Liu et al., 2025; Sun

et al., 2023; Wolfers et al., 2020). The present threshold represents a principled intermediate choice that balances sensitivity to meaningful deviations against specificity.
We nonetheless agree that the choice of a specific threshold is arbitrary. For this precise reason, we report supplementary analyses at a more lenient and more stringent threshold ($p < 0.001$ and $p < 0.05$). These analyses reveal a consistent pattern of results. Notably, at the most stringent threshold ($p < 0.001$), significant overlap in negative deviations is no longer detected at the network level, and regional overlap is markedly reduced; positive deviation overlap is also diminished. We believe this pattern is informative: rather than suggesting that our primary findings are unreliable, it indicates that very stringent thresholds may sacrifice sensitivity to the point of obscuring genuine signal. The consistency of spatial patterns across thresholds, combined with the expected attenuation at more extreme cutoffs, supports $|Z| > 2.3$ as an appropriate balance between sensitivity and specificity for characterizing connectivity heterogeneity.

We have clarified the rationale for our threshold selection in the revised Methods section.

> "Extreme deviations were defined as $|Z| > 2.3$, corresponding to approximately $p < 0.01$. Thresholds for defining extreme deviations in normative modeling studies have typically ranged from $|Z| > 1.96$ ($p < 0.05$)(Bayer et al., 2022; Lv et al., 2021) to $|Z| > 2.6$ ($p < 0.005$) (Liu et al., 2025; Sun et al., 2023; Wolfers et al., 2020). The present threshold represents a principled intermediate choice that balances sensitivity to meaningful deviations against specificity. Given that a central aim of this study is to characterize heterogeneity in autism and capture the diversity of individual-level atypical connectivity patterns, we favoured a threshold that maintains sensitivity rather than a more stringent cutoff that risks obscuring genuine variability. To ensure robustness, we report supplementary analyses at $|Z| > 1.96$ ($p < 0.05$), $|Z| > 2.6$ ($p < 0.005$), and $|Z| > 3.1$ ($p < 0.001$), which demonstrate consistent spatial patterns with the expected attenuation at more extreme thresholds (Supplement Section 9, Figures S6 and S7)."

We have also performed the analysis with the threshold used in previous literature cited by the Reviewer. This analysis is now reported on Figure S7 of the supplement. Importantly, at this threshold the key findings are consistent.

We have added the following text in the supplement:

> "Additionally, we repeated the overlap analysis using a threshold of $|Z| > 2.6$, which has been used in previous normative modeling studies of autism (Liu et al., 2025; Sun et al., 2023; Wolfers et al., 2020). The spatial pattern of group differences at both region and network levels was very similar to that obtained with our primary threshold of $|Z| > 2.3$, confirming that our findings are not driven by the specific threshold choice (Figure S7)."

Redacted

Figure S7. Threshold sensitivity analysis at |Z| > 2.6.
Overlap of extreme deviations between autistic and neurotypical groups using the threshold of |Z| > 2.6, as employed in previous normative modeling studies.

We have also added the following text to the Limitations section (page 17 of the revised manuscript):

> “Our primary analysis focused on a threshold of Z=|2.3| for defining extreme deviations. This choice is somewhat arbitrary. Our supplementary analyses indicated that our main findings are robust to the use of both more lenient and more stringent thresholds (see Supplement section 9). Future work may explore alternative, threshold-free methods for quantifying deviation heterogeneity across people.”

Results
Comment 5: Line 207: I am confused about this statement “These findings indicate that autism is associated with expected levels of extreme FC deviations when examined at the level of specific pairwise connections.” How can authors get this finding from the results?

Response: We apologise for a lack of clarity. Our intention was to state that, at the level of individual pairwise connections, autistic individuals did not show a significantly greater proportion of extreme deviations than controls. In other words, the rate of extreme deviations in the autistic group was within the range expected from the normative model. It is only when these sparse, individually idiosyncratic deviations are aggregated at the regional and network level that a coherent pattern distinguishing autistic individuals from controls emerges. We have revised the wording to make this clearer.

> "These findings indicate that, at the level of individual pairwise connections, the proportion of extreme FC deviations in autistic individuals was within the normative range (i.e., it did not significantly differ from controls), with no single connection deviating in more than approximately 4% of participants in either group."

Comment 6: Line 246-254: What about the correlations between the true and predicted values if combined the features of three levels.

Response: We appreciate this suggestion. The goal of our prediction analysis was specifically to assess the predictive value of each spatial scale independently and to understand whether connection-, region-, or network-level deviations carry distinct information about clinical and behavioral phenotypes. Combining features across levels would obscure this, as it would no longer be possible to attribute predictive performance to a specific scale. Additionally, the large difference in dimensionality across levels, particularly the connection-level features, would risk the higher-dimensional features dominating the combined model. We agree that multi-level integration is an interesting direction and have noted this in the revised discussion.

> "Future work could explore the integration of deviation features across spatial scales using methods suited to combining feature sets of different dimensionality,"

References:

Bayer, J. M. M., Dinga, R., Kia, S. M., Kottaram, A. R., Wolfers, T., Lv, J., . . . Marquand, A. (2022). Accommodating site variation in neuroimaging data using normative and hierarchical Bayesian models. *Neuroimage, 264*, 119699. doi:10.1016/j.neuroimage.2022.119699

Liu, Q., Lai, H., Le, J., Lan, C., Zhang, X., Huang, L., . . . Zhao, W. (2025). Identifying brain functional subtypes and corresponding task performance profiles in autism spectrum disorder. *Molecular psychiatry, 30*(11), 5034-5044. doi:10.1038/s41380-025-03086-x

Lv, J., Di Biase, M., Cash, R. F. H., Cocchi, L., Cropley, V. L., Klauser, P., . . . Zalesky, A. (2021). Individual deviations from normative models of brain structure in a large cross-sectional schizophrenia cohort. *Molecular psychiatry, 26*(7), 3512-3523. doi:10.1038/s41380-020-00882-5

Marquand, A. F., Kia, S. M., Zabihi, M., Wolfers, T., Buitelaar, J. K., & Beckmann, C. F. (2019). Conceptualizing mental disorders as deviations from normative functioning. *Molecular psychiatry, 24*(10), 1415-1424.

Segal, A., Parkes, L., Aquino, K., Kia, S. M., Wolfers, T., Franke, B., . . . Fornito, A. (2023). Regional, circuit and network heterogeneity of brain abnormalities in psychiatric disorders. *Nature neuroscience, 26*(9), 1613-1629. doi:10.1038/s41593-023-01404-6

Sun, X., Sun, J., Lu, X., Dong, Q., Zhang, L., Wang, W., . . . Xia, M. (2023). Mapping Neurophysiological Subtypes of Major Depressive Disorder Using Normative Models of the Functional Connectome. *Biol Psychiatry, 94*(12), 936-947. doi:10.1016/j.biopsych.2023.05.021

Wolfers, T., Beckmann, C. F., Hoogman, M., Buitelaar, J. K., Franke, B., & Marquand, A. F. (2020). Individual differences v. the average patient: mapping the heterogeneity in ADHD using normative models. *Psychological Medicine, 50*(2), 314-323.

Zabihi, M., Oldehinkel, M., Wolfers, T., Frouin, V., Goyard, D., Loth, E., . . . Dumas, G. (2019). Dissecting the heterogeneous cortical anatomy of autism spectrum disorder using normative models. *Biological Psychiatry: Cognitive Neuroscience and Neuroimaging, 4*(6), 567-578.

Reviewer #1 (Remarks to the Author):

I would like to congratulate the authors on an excellent paper and well done response. I have no further major comments.

Response: We thank the reviewer for their positive assessment and their constructive comments throughout the review process.

I found a minor typo:

Page 8 , line 228
“region level, , deviation degree”

Response: This typo has now been corrected in the revised manuscript.

Also the code git repo needs a readme explaining how to run the analysis. And while some code was shared it represents only a fraction of the analysis, has minimal comments, and loads data in an unknown format and thus is hard to read and replicate. Though some of the process is clear (ish) as the code is not especially dense (simple is good).

Response : The repository has been expanded to include: (1) a README file with instructions regarding the analysis pipeline, (2) additional scripts that generate the intermediate data structures required as input to the main analyses, and (3) more extensive inline comments throughout the code. We hope these additions make the workflow easier to follow and replicate.

I look forward to seeing future work in this direction.

Response: We thank the reviewer once again for their encouraging comments.

Repeated from author comments:

Also the code git repo needs a readme explaining how to run the analysis. And while some code was shared it represents only a fraction of the analysis, has minimal comments, and loads data in an unknown format and thus is hard to read and replicate. Though some of the process is clear (ish) as the code is not especially dense (simple is good).

Its very bare bones and only some of the calculations for outcomes.

Response: The repository has been expanded with a README detailing how to run the analysis, additional code for generating the data in the format required by the pipeline,

and more comprehensive commenting throughout. We hope the revised repository now provides a clear and complete basis for running the analyses.

Reviewer #2 (Remarks to the Author):

I appreciate that the authors have addressed all of my comments carefully. I am happy to endorse the publication of this work in its current form. Congratulations to this beautiful work!
The code is neat and sufficient to generate the reproducible pipelines.

Response: We thank the reviewer for their constructive comments and positive assessment throughout the review process.

Reviewer #3 (Remarks to the Author):

The authors have addressed all my concerns.

The current code is sufficient for reproducing these results in the manuscript.

Response: We appreciate the reviewer's constructive comments and positive assessment throughout the review process.
